# RING finger protein 5 is a key anti-FMDV host factor through inhibition of virion assembly

**DOI:** 10.1371/journal.ppat.1012848

**Published:** 2025-01-17

**Authors:** Wei Zhang, Weiwei Li, Yang Yang, Weijun Cao, Wenhua Shao, Mengyao Huang, Jiali Wang, Zhitong Chen, Jiantao Cai, Hongyi Liu, Xiaoyi Zhao, Xingyan Dong, Tingting Zhou, Hong Tian, Zixiang Zhu, Fan Yang, Haixue Zheng

**Affiliations:** 1 State Key Laboratory for Animal Disease Control and Prevention, College of Veterinary Medicine, Lanzhou University, Lanzhou Veterinary Research Institute, Chinese Academy of Agricultural Sciences, Lanzhou, China; 2 Gansu Province Research Center for Basic Disciplines of Pathogen Biology, Lanzhou, China; Stanford University, UNITED STATES OF AMERICA

## Abstract

Foot-and-mouth disease virus (FMDV) are small, icosahedral viruses that cause serious clinical symptoms in livestock. The FMDV VP1 protein is a key structural component, facilitating virus entry. Here, we find that the E3 ligase RNF5 interacts with VP1 and targets it for degradation through ubiquitination at the lys200 of VP1, ultimately inhibiting virus replication. Mutations at this lysine site have been found to increase the replication of FMDV in mice. Importantly, the RNF5 pharmacological activator Analog-1 alleviates disease development in a mouse infection model. Furthermore, RNF5 recognizes the VP1 protein from several picornaviruses, suggesting that targeting RNF5 may be a broad-spectrum antiviral strategy. These findings shed light on the role of the ubiquitin-proteasome system in controlling virus replication, offering potential new strategies for treating viral infections.

## Author summary

Elucidation of the host factors that modulate the FMDV lifecycle is vital for developing antiviral drugs and vaccines. In this study, we show that RNF5 acts as a cell-intrinsic antiviral effector by inhibiting multiple picornaviruses through targeting the viral VP1 protein for ubiquitination and subsequent degradation by the proteasome. Further investigation revealed that the Lys200 of VP1 is a specific site for RNF5-mediated ubiquitination. Disruption of this ubiquitination site led to increased replication of FMDV both *in vitro* and *in vivo*. The use of Analog-1, a pharmacological activator of RNF5, was shown to mitigate disease progression in a mouse infection model. These findings unveil a strategy for targeting picornavirus replication and offer insights that could aid in the development of new therapeutics for picornavirus infections.

## Introduction

Foot-and-mouth disease (FMD) is a highly contagious disease of the domestic and wild cloven-hooved animal species. It is caused by the FMD virus (FMDV), belongings to the genus *Aphthovirus* of the family *Picornaviridae* [1–3]. The FMDV capsid has an 8.5-kilobase, positive-sense, single-stranded RNA genome surrounded by an icosahedral capsid made up of 60 copies of each of the four structural proteins [4]. The FMDV genome encodes a polyprotein precursor that can be further proteolytically cleaved into four structural proteins (VP4, VP2, VP3, and VP1) and eight non-structural proteins (L^pro^, 2A, 2B, 2C, 3A, 3B, 3C^pro^, and 3D^pol^) [5]. These proteins by themselves cannot complete the virus’s entire lifecycle and must rely on host proteins. Host proteins have diverse types and functions. Some host proteins have antiviral properties that hinder virus replication, while others are exploited by the virus to aid in its growth. When the virus enters cells, the host antiviral system is promptly activated to suppress its replication and eliminate it. In order to maintain host adaptation, viruses have evolved strategies to manipulate the host and evade antiviral responses. Therefore, understanding virus-host interactions is crucial for studying host defense and viral disease development [6].

The VP1 protein of FMDV serves as the primary capsid protein, playing a crucial role in viral antigenicity and facilitating its entry into host cells through receptor-mediated mechanisms [7]. The G-H loop of FMDV VP1 is particularly significant in the pathogenesis of the virus, with its conserved Arg-Gly-Asp (RGD) motif serving as a recognition sequence for four integrin receptors [8–13]. Moreover, VP1 is characterized by high polymorphism and contains numerous neutralizing antigenic sites [14]. Therefore, understanding the molecular mechanism of the interactions between VP1 proteins and host cellular proteins is essential for the development of more effective therapeutic strategies. In this study, RNF5 has been identified as a potential inhibitor of multiple picornaviruses by targeting the VP1 protein.

The RING-finger protein family (RNF) has been shown to regulate antiviral responses [15,16]. RNF5, a membrane-bound E3 RING-finger ubiquitin ligase, is involved in ER-associated protein degradation (ERAD), cell motility, and the negative regulation of autophagy and ER stress [17–20]. Studies have indicated that RNF5 restricts SARS-CoV-2 replication by degrading its envelope protein E and is also exploited by viral proteins M to facilitate self-replication [21,22]. The V protein of Newcastle disease virus recruits RNF5 to polyubiquitinate MAVS, leading to MAVS degradation [23]. In this study, our research reveals that the E3 ligase RNF5 interacts with VP1 and catalyzes its ubiquitination at the K200 in VP1, resulting in its degradation by the ubiquitin-proteasome system (UPS). An *in vivo* study further confirmed that the VP1K200 mutant virus exhibited enhanced FMDV replication ability in suckling mice. Notably, RNF5 acts as a broad-spectrum antiviral effector that restricts multiple picornaviruses. Our findings suggest that RNF5-mediated VP1 protein degradation could serve as a potential therapeutic approach for treating virus infections.

## Results

### RNF5 interacts with FMDV VP1 protein

**Fig 1:**
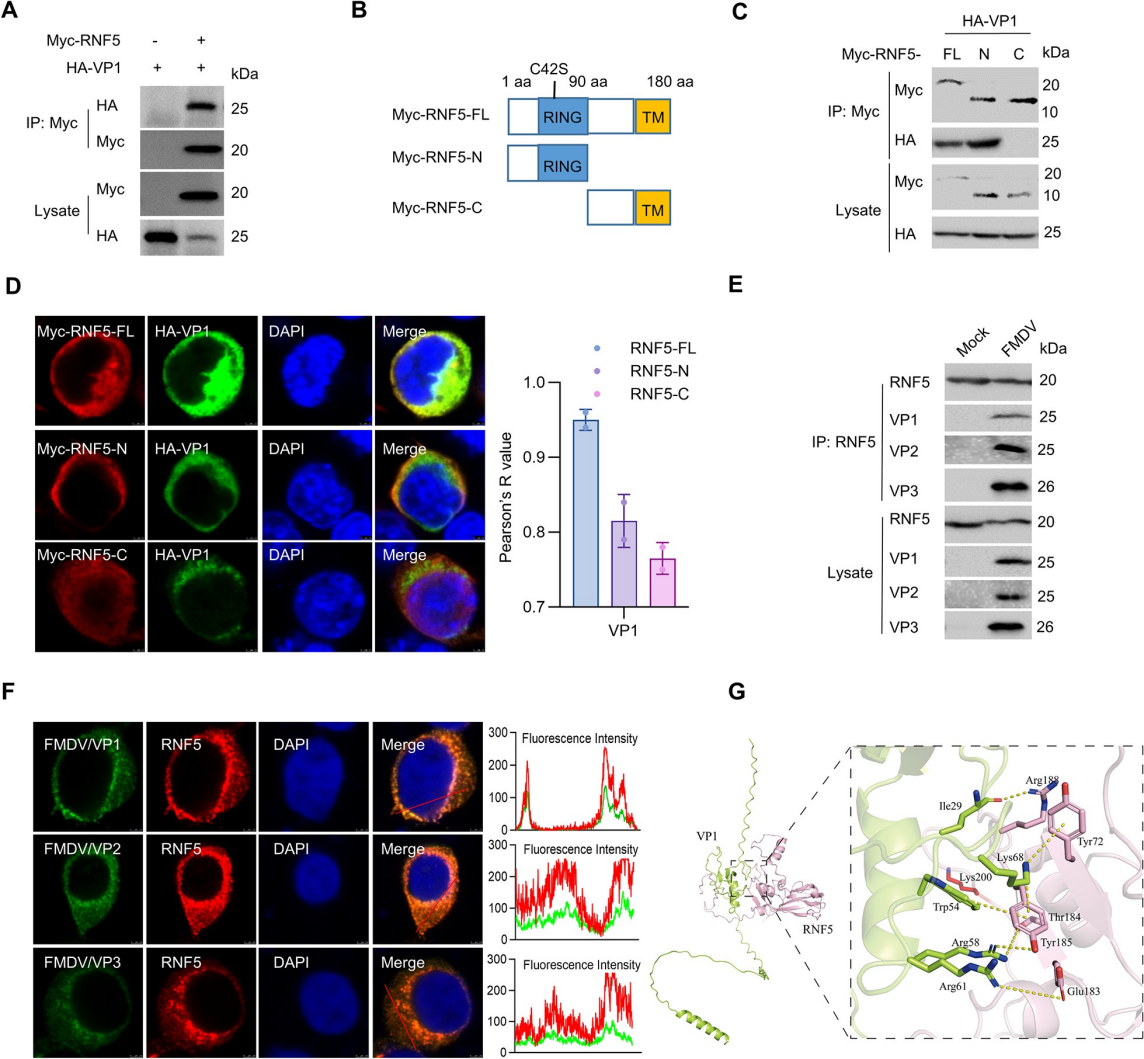
RNF5 interacts with FMDV VP1 protein. (A) Exogenous co-immunoprecipitation (Co-IP) assay in HEK293T cells. HEK293T cells were transfected with HA-VP1 and Myc-RNF5 or vector, and the cell lysates were immunoprecipitated with Myc antibody, followed by immunoblot with Myc and HA antibodies. (B) The schematic presentation of RNF5 truncated mutants. (C) The N-terminal domain of RNF5 is the main domain interaction with VP1. HEK293T cells were transfected with HA-VP1 and Myc-RNF5 or its truncations, and the cell lysates were immunoprecipitated with Myc antibodies, followed by immunoblot with Myc and HA antibodies. (D) Colocalization of VP1 with RNF5 or its truncations. HEK293T cells were transfected with HA-VP1 and Myc-RNF5 or its truncations and were then analyzed using immunofluorescence staining with anti-HA (green), anti-Myc (red), and DAPI (blue) and microscopy (Left). The co-localization analysis was expressed as Pearson’s correlation coefficient, measured for individual cells by Image J (Right). (E) Endogenous RNF5 interacted with VP1, VP2, and VP3 during FMDV infection. IBRS-2 cells were infected with or without FMDV for 12 h, and then cell lysates were immunoprecipitated with RNF5 antibodies and probed for the presence of RNF5, VP1, VP2, and VP3, respectively. (F) The colocalization of RNF5 and VP1, VP2, or VP3 in FMDV-infected cells. IBRS-2 cells were infected with FMDV for 8 h, and cells were analyzed using immunofluorescence staining with RNF5 (red), DAPI (blue), and VP1, VP2, or VP3 (green). The colocalization signals of targeted proteins were analyzed as intensity profiles of the indicated proteins by Image J line scan analysis (Right). (G) Protein interaction prediction for RNF5 (GenBank accession no. NM001123224.1) with VP1 (GenBank accession no. QID89733.1) using AlphaFold3.

It has been reported that the VP1 protein of FMDV is important for viral replication by participating in virus invasion, assembly, and release [13]. To identify host proteins that bind to VP1 of FMDV, the yeast two-hybrid system was performed. RNF5 was identified as a candidate from an unbiased screen. To verify the interaction between VP1 and RNF5, co-immunoprecipitation (Co-IP) assays were performed. The result showed that RNF5 bound to VP1 (Fig 1A). Based on the functional domain of RNF5, the protein can be categorized into two segments, namely the N-terminal and C-terminal regions. Mutants were generated for both the N-terminal and C-terminal segments of RNF5 to determine which domain is involved in the interaction with VP1 (Fig 1B). The results of the Co-IP analysis revealed that VP1 exhibited interaction with both the full-length RNF5 and its N-terminal fragments, suggesting that the N-terminal region of RNF5 serves as the primary interaction site with VP1 (Fig 1C). Additionally, the subcellular localization of the N- and C-terminal domains of RNF5 in conjunction with the VP1 protein was investigated. While RNF5 and VP1 were observed to colocalize throughout the cytoplasm, the N- and C-terminal domains of RNF5 exhibited partial colocalization with VP1 (Fig 1D). To investigate the potential interaction of RNF5 with VP1, VP2, or VP3 during FMDV infection, cellular lysates from FMDV-infected cells were subjected to immunoprecipitation. The findings revealed the formation of a complex involving RNF5, VP1, VP2, and VP3 during FMDV infection (Fig 1E). Additionally, confocal microscopy demonstrated that RNF5 could colocalize with VP1, VP2, and VP3 (Fig 1F). In addition, protein interaction prediction for VP1 with RNF5 using the AlphaFold3 to further confirm the interaction between RNF5 and VP1 (Fig 1G). In summary, these results collectively suggest that RNF5 interacts with FMDV VP1 protein.

### RNF5 inhibits FMDV replication

**Fig 2:**
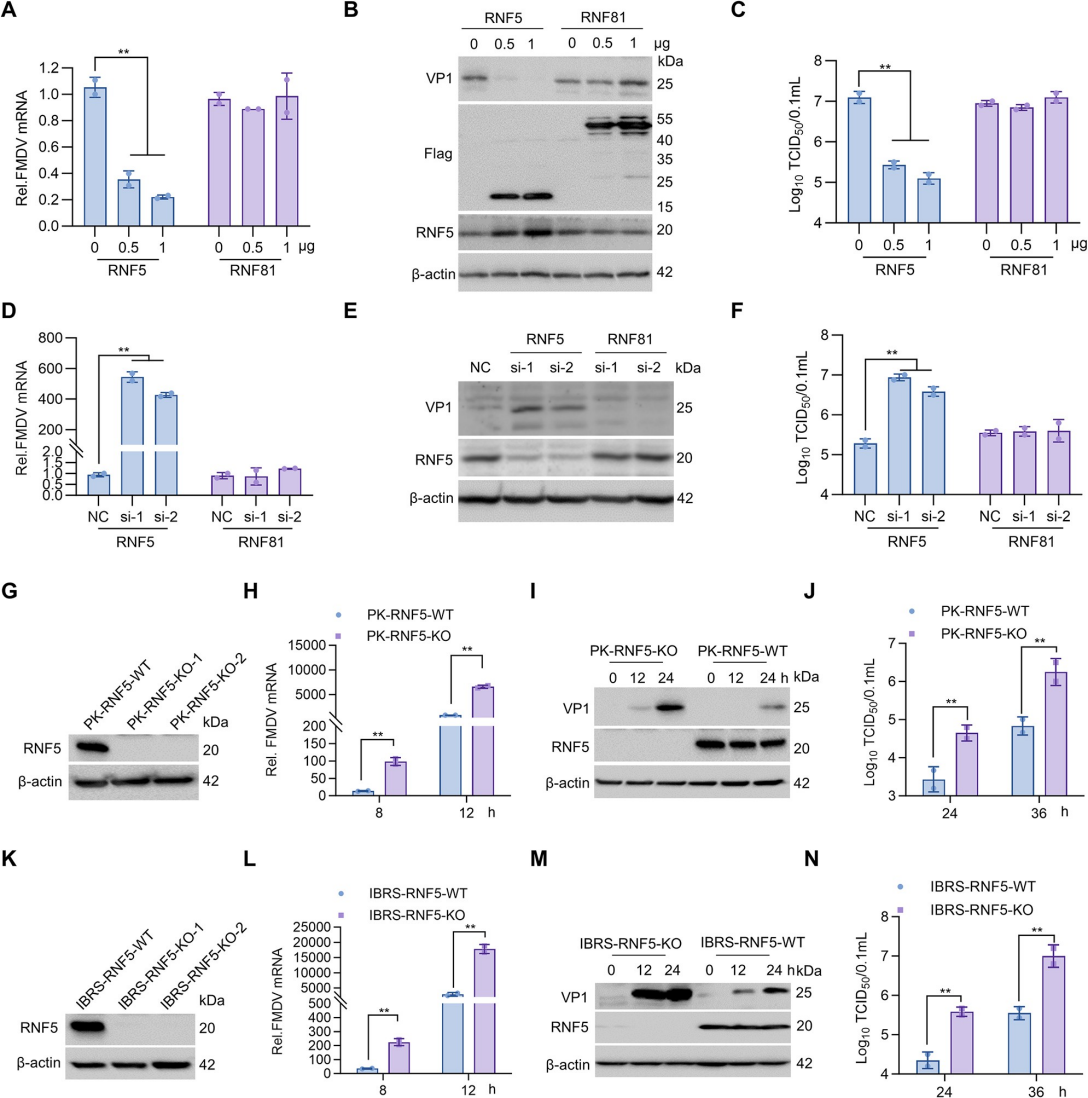
RNF5 inhibits FMDV replication. (A-C) FMDV replication was restricted by RNF5 ectopic expression. IBRS-2 cells seeded into 6-well plates were transfected with RNF5 or RNF81 plasmids in a dose-dependent manner for 24 h and then inoculated with FMDV at an MOI of 1 for 8, or 16 h. The infected cells at 8 h were harvested for detection of FMDV mRNA abundance using RT-PCR (A). The infected cells at 16 h were harvested for detection of RNF5 and FMDV VP1 protein expression using immunoblot (B), intracellular and extracellular progeny viral titers by assessing TCID_50_ (C). (D-F) Down-regulation of RNF5 promotes FMDV replication. IBRS-2 cells seeded into 6-well plates were transfected with 150 nM NC, siRNA-1, or siRNA-2 of RNF5 or RNF81 for 24 h, followed by infection with FMDV (MOI = 1) for 8 h or 16 h. The virus RNA at 8 h was extracted, and RT-PCR experiments were performed (D). The protein expression levels of endogenous RNF5 and viral VP1 proteins at 16 h were detected by immunoblot (E). FMDV yields were determined by TCID_50_ assay (F). (G) Confirmation of successful knockout of RNF5 in PK-RNF5-KO cell line by immunoblot. (H-J) Knockout of pRNF5 in PK-15 cells promotes FMDV progeny virion production. PK-RNF5-WT and PK-RNF5-KO-1 cell lines seeded into 6-well plates were infected with FMDV at 8, 12, 24, or 36 h. The mRNA expression level of FMDV was measured by RT-PCR at 8, or 12 h (H). The protein expression levels of endogenous RNF5 and viral VP1 proteins were detected by immunoblot at 12, or 24 h (I). FMDV yields were determined by TCID_50_ assay at 24, or 36 h (J). (K) Confirmation of successful knockout of RNF5 in IBRS-RNF5-KO cell lines by immunoblot. (L-N) Knockout of pRNF5 in IBRS-2 cells promotes FMDV replication. IBRS-RNF5-WT and IBRS-RNF5-KO-1 cell lines were infected with FMDV at the indicated time points. The mRNA expression level of FMDV was measured by RT-PCR (L). The protein expression levels of RNF5 and viral VP1 proteins were detected by immunoblot (M). FMDV yields were determined by TCID_50_ assay (N). Graphs show mean ± SD (n = 2 technical replicates, n = 3 biological replicates in A-N) from one representative experiment. Data were analyzed by two-way ANOVA, followed by Dunnett’s multiple comparisons test (A, C, D and F) or sidak’s multiple comparisons test (H, J, L and N). **P<0.01.

In order to investigate the impact of RNF5 on FMDV infection through its interaction with VP1 protein, IBRS-2 cells were transfected with Flag-RNF5 or RNF81 (another irrelevant RING finger protein as a control) plasmids [24], and subsequently infected with FMDV. The increased levels of RNF5 or RNF81 did not impact cell viability (S1A Fig). Viral RNA levels, viral protein abundance, and viral titers were assessed and compared using RT-PCR, immunoblot, and median tissue culture infective dose (TCID_50_) assays. Notably, RNF5 significantly inhibits FMDV mRNA levels in a dose-dependent manner, but the control protein RNF81 did not (Fig 2A). Immunoblot analysis indicated a significant reduction in the VP1 protein level due to RNF5 overexpression (Fig 2B). The viral titer also revealed a reduction in a dose-dependent manner upon RNF5 overexpression (Fig 2C). These findings suggest that the overexpression of RNF5 effectively inhibits FMDV replication.

To further investigate the impact of RNF5 knockdown on FMDV replication, three siRNAs targeting RNF5 were initially screened for knockdown efficiency. Subsequently, siRNA-1 and siRNA-2, which exhibited higher efficiency in reducing RNF5 expression, were selected for further knockdown experiments (S1B and S1C Fig). Similarly, RNF81 also designed three interfering RNAs as controls (S1D Fig). Following transfection of IBRS-2 cells with negative control (NC) siRNA, siRNA-1, or siRNA-2 of RNF5 or RNF81, and subsequent infection with FMDV, the viral RNA levels, protein expression, and titers were compared. Results from RT-PCR analysis indicated a significant increase in FMDV genome mRNA levels in RNF5 knockdown cells compared to RNF81 siRNA cells (Fig 2D). Immunoblot assays demonstrated a notable elevation in VP1 expression levels following siRNA-mediated RNF5 knockdown (Fig 2E). Furthermore, TCID_50_ assays revealed an enhancement in viral titers by approximately 1.64 log_10_ TCID_50_/0.1 mL (equivalent to around 43.65-fold) and 1.3 log_10_ TCID_50_/0.1 mL (equivalent to around 19.95-fold) respectively, in RNF5 knockdown cells (Fig 2F). These results indicate that FMDV replication levels were higher in RNF5 knockdown cells compared to RNF81 knockdown cells. These findings provide evidence that RNF5 knockdown enhances FMDV replication.

To further verify the influence of endogenous RNF5 on FMDV replication, CRISPR/Cas9 genomic editing was employed to create pRNF5 gene knockout cell lines in PK-15 and IBRS-2 cells. Two guide RNAs (gRNA-252 and gRNA-304) were utilized to target specific sites within exon 1 of the pRNF5 genome sequences (S1E Fig). The efficiency of RNF5 knockout in the cell lines was confirmed through DNA sequencing and immunoblot analysis. Sequencing of the gRNA-252 cell clone revealed a 22-nucleotide deletion in PK-15 cells (PK-RNF5-KO-1) and a one-nucleotide deletion in IBRS-2 cells (IBRS-RNF5-KO-1) near the gRNA-252 site. Additionally, sequencing of the gRNA-304 cell line identified a 2-nucleotide deletion in PK-15 cells (PK-RNF5-KO-2) and a 13-nucleotide deletion in IBRS-2 cells (IBRS-RNF5-KO-2) (S1F–S1I Fig). Cell viability assays indicated that the viability of PK-RNF5-KO-1 and PK-RNF5-KO-2 cells resembled that of wild-type PK-15 cells (S1J Fig), while IBRS-RNF5-KO-1 and IBRS-RNF5-KO-2 cells exhibited viability similar to wild-type IBRS cells (S1K Fig). Immunoblot analysis confirmed the absence of endogenous RNF5 in the established cell lines (Fig 2G and 2K). These findings demonstrate that the RNF5-KO cell lines were successfully constructed. Subsequently, in order to assess the impact of RNF5 gene knockout on FMDV replication, the wild-type (WT) and RNF5 knockout PK-15 or IBRS cell lines were infected with FMDV. Samples were collected at the indicated times. The levels of viral RNA, protein abundance, and titers were evaluated, respectively. In PK-15 cells, the absence of RNF5 resulted in a significant increase in FMDV genome mRNA (Fig 2H). Furthermore, the expression of endogenous VP1 in FMDV-infected PK-15 cells were notably elevated in the absence of RNF5 at 12 and 24 hpi (Fig 2I). The viral titer in RNF5 knockout PK-15 cells exhibited approximately 1.5 log_10_ TCID_50_/0.1 mL (equivalent to approximately 31-fold) and 1.3 log_10_ TCID_50_/0.1 mL (equivalent to approximately 19.95-fold) increases at 24 and 36 hpi, respectively (Fig 2J). Indirect immunofluorescence assay (IFA) demonstrated increased fluorescence in RNF5 knockout PK-15 cells compared to wild-type cells (S1L Fig). Similarly, in RNF5 knockout IBRS cell lines, the levels of viral RNAs, proteins, and titers were significantly elevated compared to wild-type cells (Fig 2L–2N). Altogether, gain-of-function and loss-of-function assays indicating RNF5 plays a restrictive role in FMDV replication.

### RNF5 degrades FMDV VP1 protein and affects virion assembly

**Fig 3:**
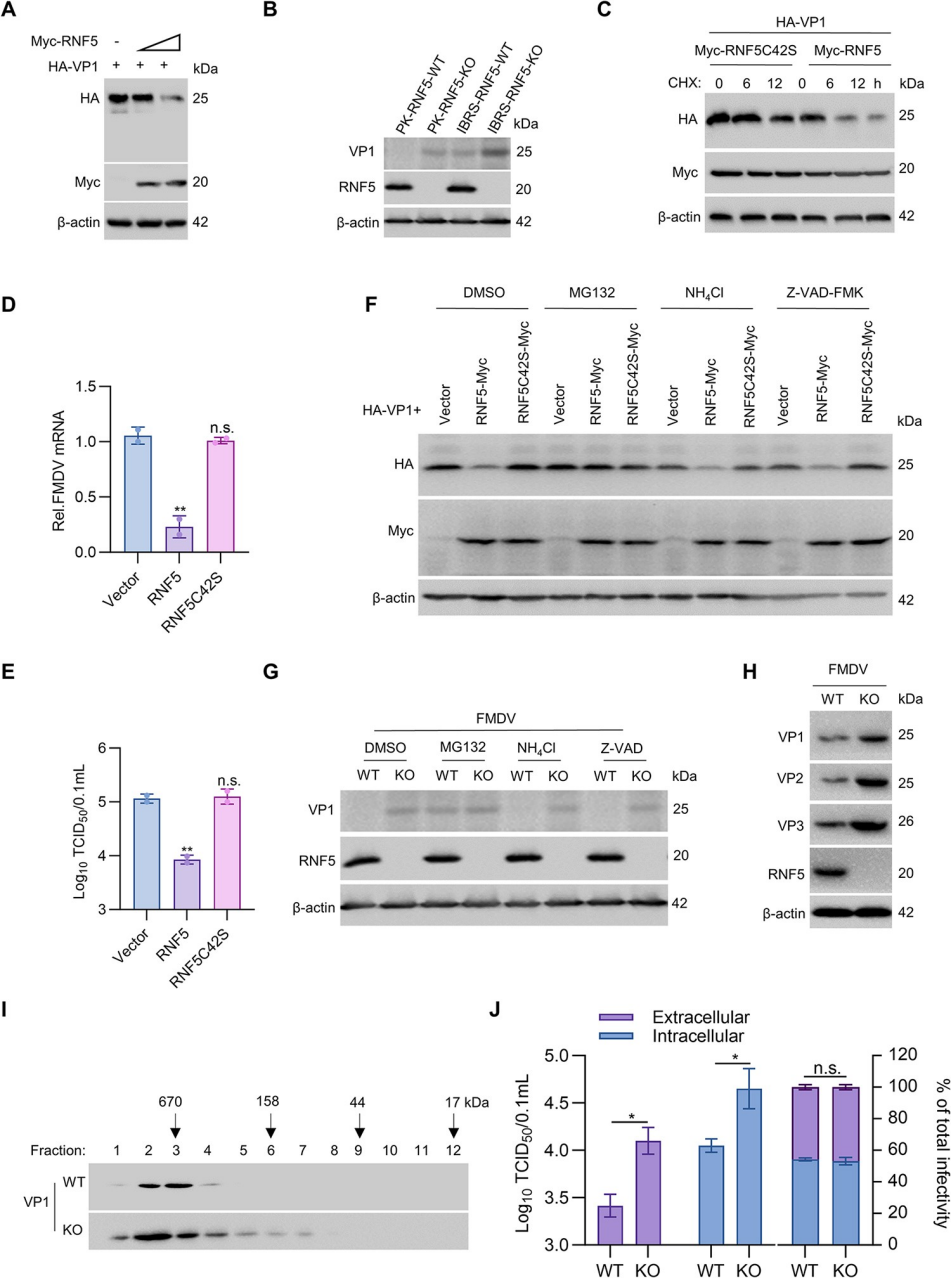
RNF5 degrades FMDV VP1 protein and affects virion assembly. (A) RNF5 induces the reduction of VP1 in a dose-dependent manner. HEK293T cells were transfected with HA-VP1 plasmid and increased quantities of Myc-RNF5 plasmids. The expression of HA-VP1 and Myc-RNF5 was detected by immunoblot. (B) The effect of endogenous RNF5 on VP1 during FMDV infection. PK-RNF5-KO, IBRS-RNF5-KO, or WT cells were infected with FMDV (MOI = 0.5) for 24 h, and then endogenous RNF5 and VP1 were detected using immunoblot. (C) RNF5 regulates the half-life of VP1 protein. HEK293T cells were co-transfected with HA-VP1 and Myc-RNF5 or Myc-RNF5C42S plasmids. After 16 h, the cells were treated with CHX (100 μg/ml) for 0, 6, and 12 h before immunoblot. (D) The mRNA expression level of FMDV was detected by RT-PCR. PK-15 cells were transfected with RNF5 or RNF5C42S plasmids and then infected with FMDV (MOI = 0.5) for 24 h. (E) FMDV titers were determined by TCID_50_ assay. PK-15 cells were transfected with RNF5 or RNF5C42S plasmids and then infected with FMDV (MOI = 0.5) for 36 h. TCID_50_ assays were performed in BHK-21 cells. (F) RNF5 promotes proteasomal degradation of VP1. HA-VP1 was transfected with Myc-RNF5 or Myc-RNF5C42S in HEK293T cells and then treated with DMSO (negative control), MG132 (20 μM), NH4Cl (20 mM), or Z-VAD-FMK (50 μM). (G) The degradation pathway of endogenous RNF5 on FMDV VP1. PK-RNF5-KO or WT cells were infected with FMDV (MOI = 0.5) for 12 h and then treated with DMSO (negative control), MG132 (20 μM), NH_4_Cl (20 mM), or Z-VAD-FMK (50 μM) for another 12 h. The endogenous RNF5 and VP1 proteins were detected by immunoblot. (H) The effect of endogenous RNF5 on VP1, VP2, or VP3 during FMDV infection. IBRS-RNF5-KO, or WT cells were infected with FMDV (MOI = 1) for 12 h, and then endogenous RNF5 and VP1, VP2, or VP3 were detected using immunoblot. (I) The effects of RNF5 on the assembly intermediates of virion. WT and KO cells were infected FMDV for 12 h. Cells were collected and lysed, followed by gel filtration. Fraction size was calibrated with the gel filtration standard (151–1901, Bio-Rad). (J) The effects of RNF5 on the virus release. WT or KO cells were infected with FMDV at an MOI of 1 for 12 h, the cells and supernatants were harvested to detect extracellular virus titers and the intracellular virus titers (Left). The efficiency of virus secretion was determined as the ratio of extracellular and intracellular infectivity. Graphs show mean ± SD (n = 2 technical replicates, n = 3 biological replicates in D, E and J) from one representative experiment. Data were analyzed by one-way ANOVA with Dunnett’s multiple comparisons test (D and E) or two-way ANOVA with sidak’s multiple comparisons test (J). **P<0.01; n.s., indicating no statistical significance.

To further explore the underlying molecular processes of RNF5 in inhibiting FMDV, subsequent analyses were conducted. Specifically, dose-response experiments were carried out to assess the impact of RNF5 on the expression of VP1 protein. Plasmids containing HA-VP1 with a HA tag at the carboxyl-terminal and Flag-VP1 with a Flag tag at the amino-terminal were employed to validate the decrease in VP1 levels induced by RNF5. The results indicated a reduction in VP1 protein levels upon RNF5 expression in a dose-dependent manner, with no evidence of cleaved bands observed. The relative fold-change in VP1 protein abundance was assessed through densitometric analysis utilizing ImageJ Launcher, revealing that RNF5 notably degraded VP1 protein (Figs 3A and S2A–S2C). Nevertheless, the overexpression of RNF5 did not lead to a significant reduction in VP1 mRNA levels (S2D Fig). Additionally, the impact of endogenous RNF5 on VP1 during FMDV infection was investigated. PK-RNF5-KO or IBRS-RNF5-KO cells were infected with FMDV, and endogenous RNF5 and VP1 proteins were detected. The outcomes indicated that PK-RNF5-KO and IBRS-RNF5-KO cells exhibited a marked increase in VP1 expression during FMDV infection compared to wild-type cells (Fig 3B). These findings suggest that RNF5 diminishes FMDV VP1 expression at the protein level.

RNF5 is an enzyme characterized by a RING finger domain with E3 ubiquitin ligase activity [25]. In this investigation, RNF5C42S, a variant of RNF5 with a mutated Cys42 in the ring-finger domain to serine and lacking E3 ubiquitin ligase activity, was utilized to examine the necessity of the E3 ubiquitin ligase activity of RNF5 for FMDV inhibition. To further confirm the suppressive activity of RNF5 on VP1 expression, the half-life of the VP1 protein was tested. VP1 and RNF5C42S or RNF5 plasmids were co-transfected into 293T cells and then treated with CHX (a specific inhibitor of protein synthesis) for another 0, 6, 12 h. The result showed that control to RNF5C42S, RNF5 significantly accelerated the degradation of VP1 in cells treated with CHX, indicating RNF5 controls the half-life of VP1(Fig 3C). Subsequently, the study investigated the impact of RNF5 enzyme activity sites on the inhibition of FMDV. To conduct this investigation, PK-15 cells were transfected with either vector, RNF5, or RNF5C42S plasmids, followed by infection with FMDV. The results obtained from RT-PCR and TCID_50_ assays indicated that RNF5 significantly suppressed FMDV replication, whereas RNF5C42S did not exhibit any inhibitory effect. This observation suggests that enzyme active sites play a crucial role in inhibiting the replication of FMDV (Fig 3D and 3E).

The degradation of proteins typically involves three major pathways, namely proteasome, autophagy-lysosome, and caspase pathways. In this study, cells were treated with specific inhibitors, including MG132 for proteasome, NH_4_Cl for lysosome, and Z-VAD-FMK for caspase, to determine the pathway responsible for VP1 degradation. The findings revealed that only MG132 mitigated the VP1 degradation induced by RNF5, while NH_4_Cl and Z-VAD-FMK did not have any impact, indicating that RNF5 degraded VP protein through the proteasome pathway (Fig 3F). Furthermore, the investigation delved into the degradation pathway of endogenous RNF5 on FMDV VP1. PK-RNF5-KO or WT cells were infected with FMDV and subsequently treated with DMSO, MG132, NH_4_Cl, or Z-VAD-FMK, followed by the detection of endogenous RNF5 and VP1. The results demonstrated that only MG132 restored the endogenous RNF5-induced VP1 degradation, while NH_4_Cl and Z-VAD-FMK did not exhibit any effect, indicating that endogenous RNF5 also degrades VP1 protein through the proteasome pathway (Fig 3G). Due to the complex formation of structural proteins VP1, VP2, and VP3 under viral infection, we conducted immunoblot experiments to investigate the effects of RNF5 on the expression of VP2 and VP3 proteins. The results showed that RNF5-KO cells increased the VP2 and VP3 expression during the process of viral infection compared to WT cells, indicating that RNF5 also mediated the degradation of VP2 and VP3 (Fig 3H). These findings collectively suggest that RNF5 triggers the degradation of FMDV VP1 proteins via the proteasome-dependent pathway, with the enzyme active site of RNF5 playing a pivotal role in inhibiting FMDV replication.

We next assessed which step in the viral replication cycle is targeted by RNF5. Initially, RNF5-KO and wild-type (WT) cells were exposed to FMDV and incubated at 4°C for 1 hour to allow viral adsorption. Subsequently, unbound viruses were removed, and the levels of cell-bound FMDV RNA was examined, revealing no difference (S2E Fig). Following this, the role of RNF5 in FMDV internalization was explored by exposing RNF5-KO and WT cells to FMDV at 4°C for 1 hour and then at 37°C for an additional hour to facilitate internalization. Analysis through RT-PCR indicated that RNF5 did not influence FMDV internalization (S2F Fig). Building upon these findings, the study investigated whether RNF5 might modulate mRNA translation or viral RNA synthesis. A bicistronic reporter plasmid was employed to assess FMDV internal ribosome entry site (IRES) activity, where the translation of the first cistron (Renilla luciferase [Rluc]) is cap-dependent, while the translation of the second cistron (firefly luciferase gene [Fluc]) relies on FMDV IRES activity (S2G Fig). The relative IRES activity was quantified as the ratio of Fluc expression to Rluc expression. The bicistronic reporter plasmid was transfected into RNF5-KO and WT cells, and after 36 hours post-transfection, cell lysates were collected to determine the Fluc activity to Rluc activity ratio. The results indicated no alteration in FMDV IRES activity between RNF5-KO and WT cells (S2H Fig). During FMDV infection, the genomic RNA serves as the template for both translation and RNA replication, thereby closely linking these processes [26]. To assess the impact of RNF5 on viral RNA synthesis, the presence of negative-strand and positive-strand viral RNA (-vRNA and +vRNA) was determined using RT-PCR. Comparative analysis between RNF5 knockout and wild-type cells revealed no significant disparity in the production of +vRNA and–vRNA within 4 h, indicating RNF5 does not affect FMDV synthesis (S2I and S2J Fig). After 6 hours, there were notable differences in the positive and negative sense viral RNAs between WT and KO cells, likely because RNF5 affects the production of new viral particles.

Building upon these results, it was postulated that RNF5 may be involved in the assembly or release of FMDV. To investigate this hypothesis, the effects of RNF5 on the assembly intermediates of virion was tested by gel filtration chromatography. The result showed that knocking out RNF5 promoted the formation of virion assembly intermediates (Fig 3I). Furthermore, both the cells and the supernatants were gathered following infection with FMDV. The results of the titer determination indicated that the knockout of RNF5 led to an increase in the production of both intracellular and extracellular progeny viruses. However, there was no difference in the intracellular and extracellular viral quantities ratio to total quantities, suggesting that RNF5 does not affect virus release (Fig 3J). Altogether, these results indicate that RNF5 primarily affect virion assembly stage of the FMDV lifecycle.

### RNF5 catalyzes the K48-linked VP1 polyubiquitination

**Fig 4:**
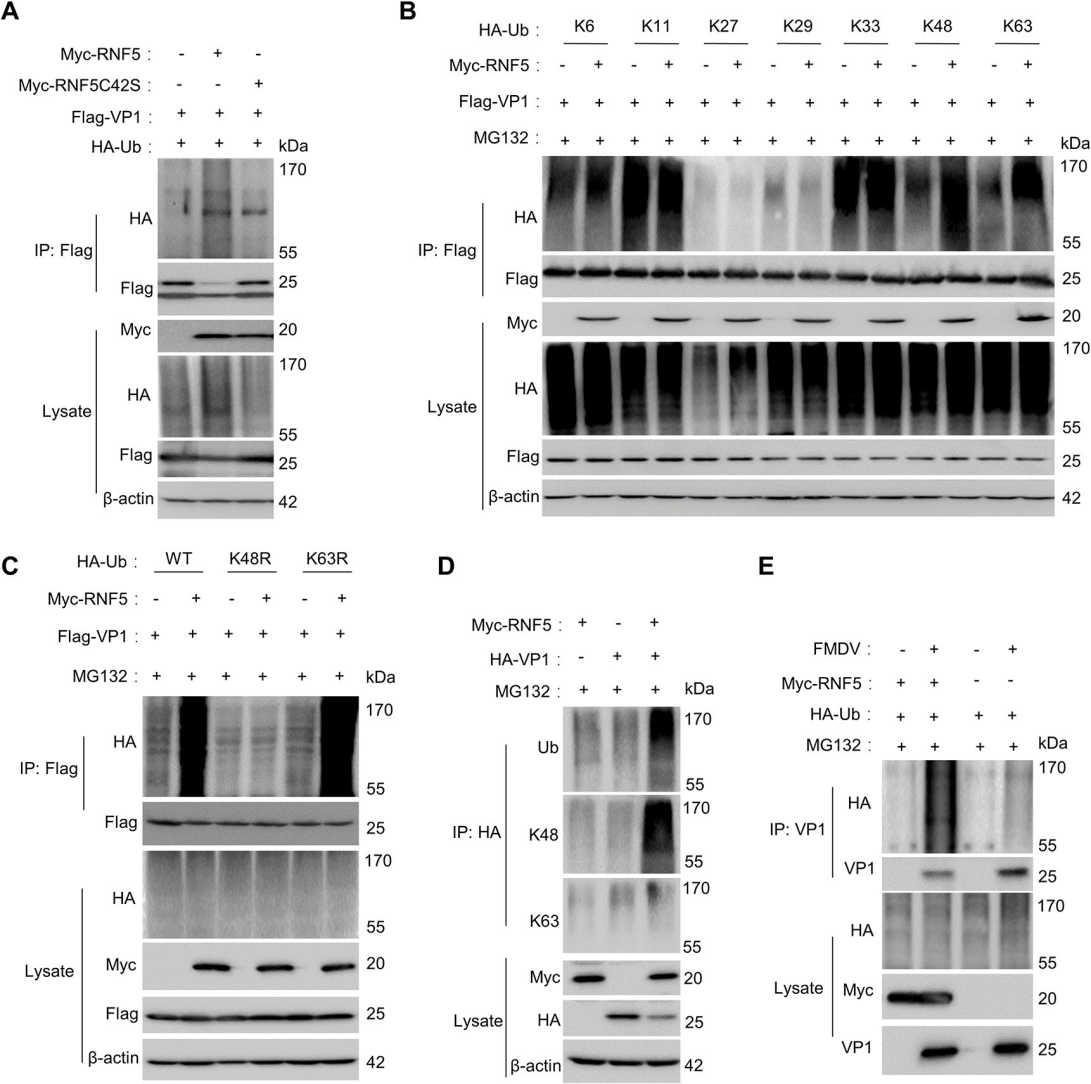
RNF5 catalyzes the K48-linked polyubiquitination of VP1. (A) RNF5 increased the ubiquitination of VP1. Flag-VP1 and Myc-RNF5 or Myc-RNF5C42S were co-transfected into HEK293T cells with HA-Ub, and then treated with MG132 (20 μM). VP1 was immunoprecipitated with Flag antibody, and ubiquitination of VP1 was detected with HA antibody. (B and C) RNF5 catalyzes the K48-linked VP1 polyubiquitination. HEK293T cells were co-transfected with Myc-RNF5, Flag-VP1 and HA-Ub wild-type (WT) or mutants (K6, K11, K27, K29, K33, K48, K63, K48R, or K63R), and then treated with MG132 (20 μM). VP1 was immunoprecipitated with Flag antibody, and ubiquitination of VP1 was detected with HA antibody. (D) Co-immunoprecipitation analysis of endogenous ubiquitination of VP1. Myc-RNF5 and HA-VP1 were co-transfected into HEK293T cells and then treated with MG132 (20 μM). VP1 was immunoprecipitated with HA antibody, and ubiquitination of VP1 was detected with Ub, K48, and K63 antibodies. (E) RNF5 regulated endogenous ubiquitination of VP1 upon FMDV infection. Lysates of PK-15 cells co-transfected with HA-Ub and Myc-RNF5 or empty plasmids with or without FMDV (MOI = 5) for 8 h in the presence of MG132 (20 μM) were subjected to IP with anti-VP1 antibody. Membranes blotted with antibodies against HA, VP1, and Myc.

The study examined the mechanism by which RNF5 targets VP1 for proteasome degradation through its enzyme active site, emphasizing the importance of protein ubiquitination in this pathway [25,27]. The investigation focused on determining whether RNF5 ubiquitinates VP1, with experiments conducted to assess ubiquitination modifications on VP1 following immunoprecipitation of ectopically expressed Flag-VP1. The results indicated that RNF5 significantly increased ubiquitination modifications on VP1, while RNFC42S did not, highlighting the essential role of RNF5 and its E3 ligase activity in VP1 ubiquitination (Fig 4A). In addition, we conducted immunoprecipitation experiments to investigate the effects of RNF5 on the ubiquitination of VP2 and VP3 proteins. The results showed that RNF5-KO cells inhibited their ubiquitin levels during the process of viral infection compared to WT cells, indicating that RNF5 also mediated ubiquitination of VP2 and VP3 (S3A–S3B Fig).

Various Ub mutants (K6, K11, K27, K29, K33, K48, K63, K48R, or K63R) were utilized to examine the impact of RNF5 on different types of VP1 polyubiquitination (S3C Fig), revealing that RNF5 overexpression enhanced K48-linked and K63-linked VP1 polyubiquitination (Fig 4B). Specifically, only HA-K48R-Ub impaired VP1 ubiquitination, underscoring RNF5 of mediation of K48-linked VP1 polyubiquitination (Fig 4C). Furthermore, the study detected endogenous ubiquitin conjugated to VP1 in the presence of RNF5, demonstrating that RNF5 catalyzed the endogenous ubiquitin conjugation to VP1, with a significantly higher amount of K48-linked ubiquitination observed in RNF5-expressing cells compared to control cells. Conversely, the amount of K63-linked VP1 ubiquitination did not show a significant difference between RNF5-expressing and control cells (Fig 4D). In order to investigate the ubiquitination of FMDV VP1 in FMDV-infected cells, PK-15 cells were co-transfected with Ub and RNF5 or control plasmids in the presence of MG132, with or without FMDV. The findings revealed that overexpression of RNF5 promoted the ubiquitination of VP1 in FMDV-infected cells (Fig 4E). To assess the impact of endogenous RNF5 on VP1 ubiquitination in FMDV-infected cells, RNF5-KO cells and WT cells were transfected with Ub plasmid, with or without FMDV. The outcomes indicated that compared to WT cells, the ubiquitination of FMDV VP1 was notably reduced in FMDV-infected RNF5-KO cells (S3D Fig). These results provide evidence that RNF5 facilitates the K48-linked polyubiquitination of VP1.

### Lys200 of VP1 is the ubiquitination site for RNF5

**Fig 5:**
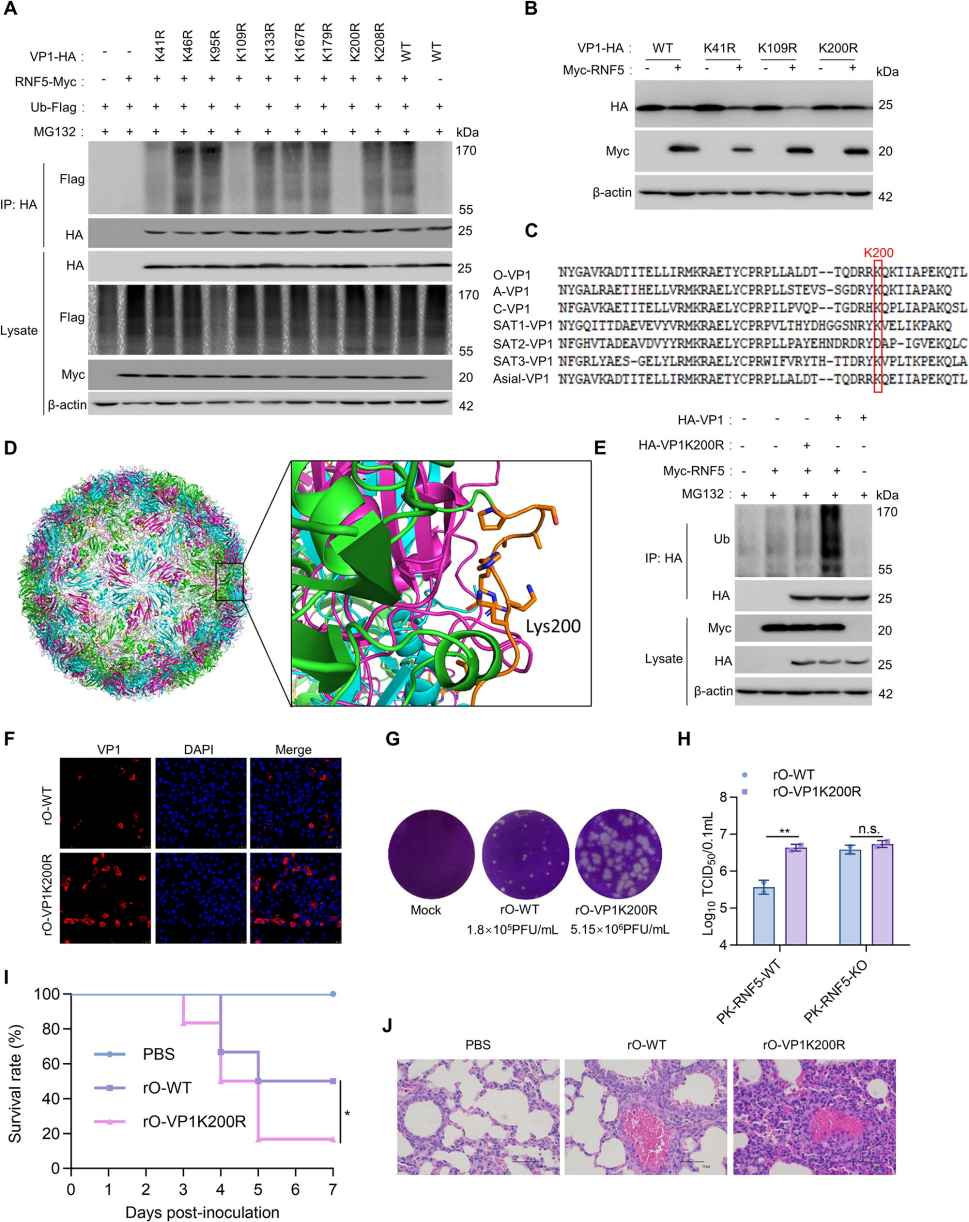
Lys200 of VP1 is the ubiquitination site for RNF5. (A) Immunoblot analysis of the ubiquitination of wildtype or mutants VP1 in HEK293T cells. Myc-RNF5, Flag-Ub, and HA-VP1 or mutants were co-transfected into HEK293T cells and then treated with MG132 (20 μM). VP1 or its mutants was immunoprecipitated with HA antibody, and ubiquitination of VP1 or its mutants was detected with Flag antibody. (B) Immunoblot analysis of the degradation of wildtype or mutants VP1 in HEK293T cells. HA-VP1 and three VP1 mutants (K41R, K109R, and K200R) were transfected with or without Myc-RNF5 for 36 h. The degradation of VP1 was visualized by immunoblot. (C) Alignment of VP1 sequences from one representative of each serotype of FMDV. Type A FMDV (GenBank accession no. QZE50273.1); Type Asia I FMDV (GenBank accession no. ACN81751.1); Type C FMDV (GenBank accession no. APX53725.1); Type O FMDV (GenBank accession no. QID89733.1); Type SAT1 FMDV (GenBank accession no. AUN44148.1); Type SAT2 FMDV (GenBank accession no. APX53730.1); Type SAT3 FMDV (GenBank accession no. APX53733.1). (D) Lys200 is on the surface of the virion. The PyMOL visualization created using the FMDV 3D structure from the Protein Data Bank (7ENP). (E) K200 attenuated the ubiquitination impact of RNF5 on VP1. HEK293T cells were co-transfected with Myc-RNF5, HA-VP1K200R, or HA-VP1 plasmids and then treated with MG132 (20 μM). VP1 or VP1K200R was immunoprecipitated with HA antibody, and endogenous ubiquitination of VP1 or VP1K200R was detected with Ub antibody. (F) Immunofluorescence analysis of rO-WT and rO-VP1K200R in BHK-21 cells. BHK-21 cells were infected with rO-WT or rO-VP1K200R at an MOI of 0.5, fixed after 12 hpi, probed with an anti-VP1 monoclonal antibody and 594-conjugated secondary antibody. (G) The plaque morphology in BHK-21 cells infected by rO-WT and rO-VP1K200R. Equal amounts (MOI = 1) of each virus were used to infect the BHK-21 cells for 8 h. The mixture of cell supernatant and sediment was repeatedly frozen and thawed three times, and then 200 μL of each mixture were serial 10-fold dilutions and used to infect the BHK-21 cells. Then, plaque phenotype and virus yield of the virus was characterized by plaque assay. (H) Viral titers of PK-RNF5-WT or PK-RNF5-KO cells infected with rO-WT or rO-VP1K200R mutant virus. PK-RNF5-WT and PK-RNF5-KO cells were infected with rO-WT or rO-VP1K200R mutant virus for 48 h and the viral titers were determined by TCID_50_ assay. (I) The survival rate of the suckling mice was monitored daily for 7 days after infection. 3-day-old suckling mice in each group of six animals were inoculated by cervicodorsal injection with PBS, rO-WT, or rO-VP1K200R (20LD_50_) in PBS, respectively. (J) Images of hematoxylin and eosin staining of lung sections from the suckling mice (3 days post-infection, n = 3 per group). Scale bars, 50 μm. Graphs show mean ± SD (n = 2 technical replicates, n = 3 biological replicates in H) from one representative experiment. Data were analyzed by two-way ANOVA with sidak’s multiple comparisons test. Survival rate of mice was examined via log-rank (Mantel-Cox) test (I, n = 6 per group, *p* = 0.0177). *P<0.05; **P<0.01; n.s., indicating no statistical significance.

E3 ubiquitin ligase mainly modifies the lysine of the target protein via ubiquitination [28]. FMDV VP1 contains nine lysine residues located in various domains. In this investigation, mutant VP1 proteins were created by substituting lysine residues with arginine at positions K41, K46, K95, K109, K133, K167, K179, K200, and K208 to determine the ubiquitination sites of VP1 by RNF5. Co-transfection of Ub, RNF5, and VP1 or its mutants was performed in 293T cells in the presence of MG132. The findings revealed that mutations at K41, K109, and K200 attenuated the ubiquitination impact of RNF5 on VP1 (Fig 5A). Subsequently, analysis through immunoblot revealed that the expression levels of VP1 WT, K41R, and K109R were notably decreased upon overexpression of RNF5. Conversely, the expression of K200R was not affected by RNF5 overexpression (Figs 5B and S3E). Subsequently, we compared the amino acid sequences of seven subtypes of FMDV. The amino acid residues at positions K200 in VP1 protein of FMDV were found to be conserved (Fig 5C). In addition, the PyMOL drawing show that Lys200 is indeed on the surface of the virion, indicating the possibility of the binding to RNF5 (Fig 5D). To further investigate the ubiquitination site of VP1, this study examined the endogenous ubiquitin conjugation to VP1 and VP1 K200R in the presence of RNF5. The findings demonstrated that RNF5 facilitated the endogenous ubiquitin conjugation to VP1, while it had no impact on the endogenous ubiquitin conjugation to K200R (Fig 5E). These outcomes indicate that RNF5 catalyzes the ubiquitination of VP1 at the Lys200 residue.

The mutant viruses containing the K200R substitution (rO-VP1K200R) were generated using reverse genetics technology [29], with the wild-type virus (rO-WT) serving as the control virus to investigate the impact of Lys200 on FMDV replication. The IFA was employed to identity the rescued viruses, indicating the successful rescue of the two viruses (Fig 5F). To compare the replication abilities and plaque phenotype of the recombinant viruses and parental virus in BHK-21 cells, plaque assays were performed. The results revealed that the recombinant virus displayed larger plaque sizes and approximately 29-fold higher viral titers compared to those produced by the parental virus (Fig 5G). Furthermore, the rO-VP1K200R mutant virus and rO-WT virus exhibited similar titers in RNF5-KO cells, but the rO-VP1K200R mutant virus displayed a higher titer than rO-WT in RNF5-WT cells, suggesting the critical role of the K200 in VP1 in reducing viral titers induced by RNF5 (Fig 5H). We next assessed pathogenic outcomes in suckling mice infected with rO-VP1K200R and rO-WT. Infection with rO-VP1K200R led to a lower survival rate compared to rO-WT (Fig 5I). Moreover, rO-VP1K200R induced severe inflammatory lung damage (Fig 5J). These results indicate that disrupting the ubiquitination site of Lys200 in VP1 can enhance the replication capabilities of FMDV both *in vitro* and *in vivo*.

### RNF5 deletion enhances the susceptibility of suckling mice to FMDV infection

**Fig 6:**
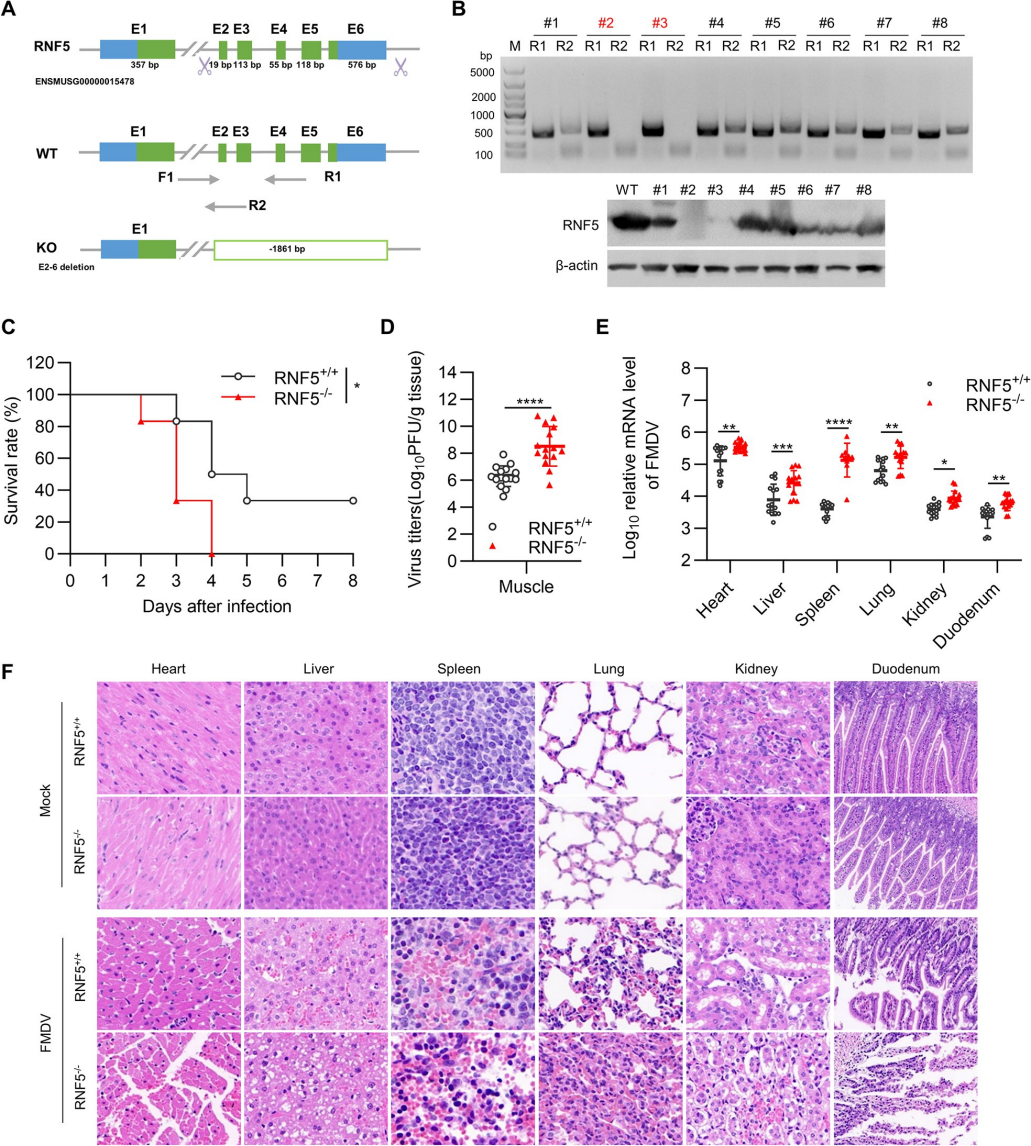
RNF5 deletion enhances the susceptibility of suckling mice to FMDV infection. (A) Knockout schematic representation position of the sequence for RNF5 in RNF5^-/-^ mice. The exon 2–6 and part of intron of RNF5 gene were deleted by CRISPR/Cas9 technology. (B) Expression and protein levels of RNF5 in RNF5^-/-^ and RNF5^+/+^ mice were detected by PCR amplification and Western blot to determine RNF5 knockout efficiency. (C) Three-day-old RNF5^-/-^ and RNF5^+/+^ suckling mice were used and injected subcutaneously with 20LD_50_ FMDV. The survival rates of all group were monitored for 8 days. (D) Viral titers in muscle of RNF5^-/-^ and RNF5^+/+^ mice challenged with FMDV. (E) FMDV mRNA levels in heart, liver, spleen, lung, kidney, and duodenum of RNF5^-/-^ and RNF5^+/+^ mice challenged with FMDV (n = 5 per group). Data were obtained from three independent experiments. (F) Images of hematoxylin and eosin staining of heart, liver, spleen, lung, kidney, and duodenum sections from the suckling mice (3 days post-infection, n = 3 per group). Scale bars, 50 μm. Survival rate of mice was examined via log-rank (Mantel-Cox) test (C, n = 6 per group, *p* = 0.029). Graphs show mean ± SD (n = 3 biological replicates, n = 5 per group in D and E). Data were analyzed by unpaired, two-tailed Studuent’s *t*-test (D) or two-way ANOVA with sidak’s multiple comparisons test (E). *P<0.05; **P<0.01; ***P<0.001; ****P<0.0001.

In order to assess the functional significance of RNF5 in FMDV infection *in vivo*, RNF5-knockout mice were generated using CRISPR/Cas9 technology. Analysis revealed that the RNF5-mutated gene exhibited a deletion spanning 1861 base pairs (Fig 6A). Subsequently, F1 mice were subjected to genotyping procedures and RNF5 protein analysis (Fig 6B). Subcutaneous administration of 20 median lethal doses (LD_50_) of FMDV was performed on both wild-type and RNF5-knockout suckling mice, with subsequent monitoring survival rates of the infected mice. The data indicated RNF5-knockout mice were more susceptible to FMDV (Fig 6C). Correspondingly, viral titers in the muscle tissues of RNF5-knockout mice were found to be roughly 100 times higher compared to those in wild-type mice (Fig 6D). Tissue samples collected from the heart, liver, spleen, lung, kidney, and duodenum of the suckling mice at 3 days postinfection were subjected to RT-PCR and hematoxylin and eosin (H&E). The study revealed that the levels of FMDV mRNA were elevated in tissues of RNF5-knockout mice compared to those of wild-type mice (Fig 6E). In comparison to the control group, wild-type and RNF5-deficient mice infected with FMDV exhibited different degrees of histopathological injuries. Notably, RNF5-deficient mice displayed more severe histopathological damages, including cardiac muscle fiber disarray, hepatocytes vacuolar degeneration, lymphocyte necrosis in the spleens, interstitial pneumonia with lymphocyte infiltration, enlarged renal glomeruli, necrosis and shedding of intestinal villi (Fig 6F). These results suggest that the depletion of RNF5 increased the replication of FMDV *in vivo*, rendering the mice higher susceptible to FMDV infection.

### RNF5 pharmacological activator Analog-1 alleviates the pathogenic effects of FMDV in a mouse model

**Fig 7:**
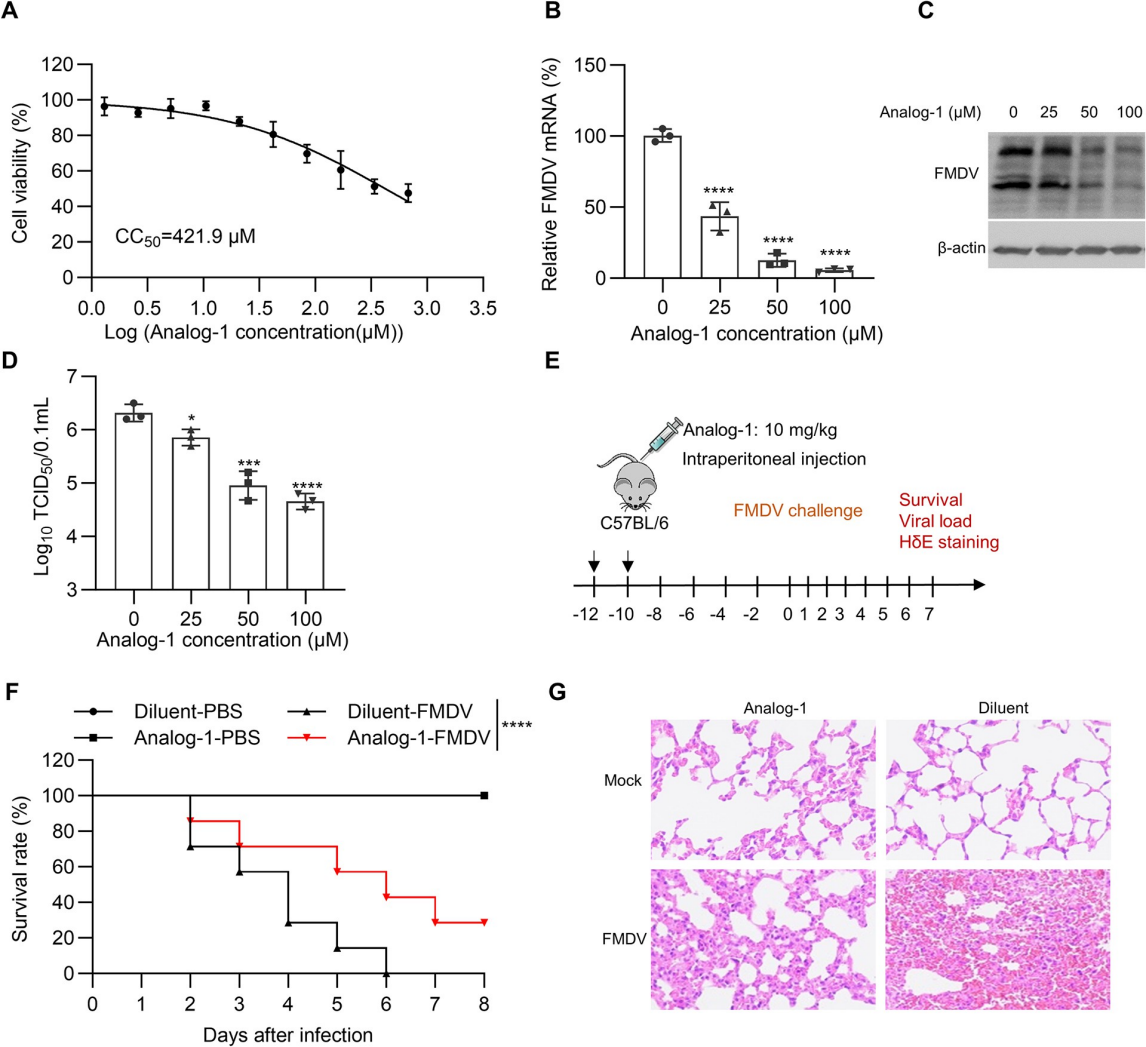
RNF5 pharmacological activator Analog-1 alleviates the pathogenic effects of FMDV in a mouse model. (A) The cell viability was measured and compared with the untreated cell control group. (B-D) PK-15 cells were exposed to varying concentrations of Analog-1 (0, 25, 50, and 100 μM) and subsequently infected with FMDV at 0.1 MOI. Following a 24-hour post-infection period, the levels of viral RNA, viral protein, and viral titers were assessed through RT-PCR (B), immunoblot (C), and TCID_50_ (D), respectively. (E) C57BL/6 mice were treated with Analog-1 at dosage of 10 mg/kg for seven times at 2-day intervals, then infected with the FMDV at dosage of 30LD_50_. (F) Survival rates of mice injected with Analog-1 after being challenged with FMDV (n = 7 per group). (G) Representative images of H&E staining of lungs of differently treated mice (3 days post-infection, n = 3 per group). CC_50_ values were calculated from nonlinear regression plots (n = 2 technical replicates, n = 3 biological replicates in A) from one representative experiment. Graphs show mean ± SD (n = 3 technical replicates, n = 3 biological replicates in B and D) from one representative experiment. Data were analyzed by one-way ANOVA with Dunnett’s multiple comparisons test (B and D). Survival rate of mice was examined via log-rank (Mantel-Cox) test (F, n = 7 per group, p<0.0001). *P<0.05; ***P<0.001; ****P<0.0001.

A recent investigation revealed that the pharmacological activator Analog-1 of RNF5 has been shown to suppress the virulence of SARS-Cov-2 by enhancing RNF5 enzymatic activity and facilitating the degradation of the E protein in a mouse model [21]. Consequently, we conducted experiments to determine the impact of this compound on the replication of FMDV. The cytotoxicity of Analog-1 was evaluated by treating PK-15 cells with 2-fold serial dilutions of Analog-1. Following a 48-hour incubation period, cell viability was assessed and compared to control cells that were not treated with the compound. The cytotoxic effect of Analog-1 on PK-15 cells was dose dependent and the concentration of cytotoxicity 50% (CC_50_) was calculated to be 421.9 μM (Fig 7A). To explore the antiviral efficacy of Analog-1 against FMDV infection, PK-15 cells were exposed to varying concentrations of Analog-1 and subsequently infected with FMDV. Following a 24-hour post-infection period, the levels of viral RNA, viral protein, and viral titers were assessed through RT-PCR, immunoblot, and TCID_50_, respectively. These findings demonstrated a notable reduction in viral RNA level (Fig 7B), viral protein level (Fig 7C), and viral titers (Fig 7D) in cells treated with different concentrations of Analog-1 compared to the untreated control group, indicating a dose-dependent antiviral activity of Analog-1 against FMDV. To confirm whether the inhibitory effect of Analog-1 on FMDV is mediated by RNF5, we investigated the effect of Analog-1 activators on the rO-VP1K200R mutant virus. The results showed that Analog-1 had no impact on the replication of the rO-VP1K200R (S4A–S4C Fig).

Subsequently, the impact of Analog-1 on FMDV virulence was evaluated using a mouse infection model. C57BL/6 mice, subjected to seven treatments of 10 mg/kg Analog-1 at 2-day intervals, were infected with FMDV at a dosage of 30LD_50_ (Fig 7E). Over an 8-day monitoring period, it was observed that all FMDV-infected groups treated with diluent succumbed to the infection within 6 days, while the survival rate in FMDV-infected Analog-1 groups was 28.57%, indicating the antiviral effects of Analog-1 (Fig 7F). Histopathological examination of the lungs revealed that treatment with Analog-1 mitigated lung lesions, such as alveolar shrinkage, typically observed in control mice (Fig 7G). Altogether, Analog-1 treatment mitigated the pathogenic effects of FMDV in a mouse model.

### RNF5 inhibits several picornavirus replication by degrading VP1

**Fig 8:**
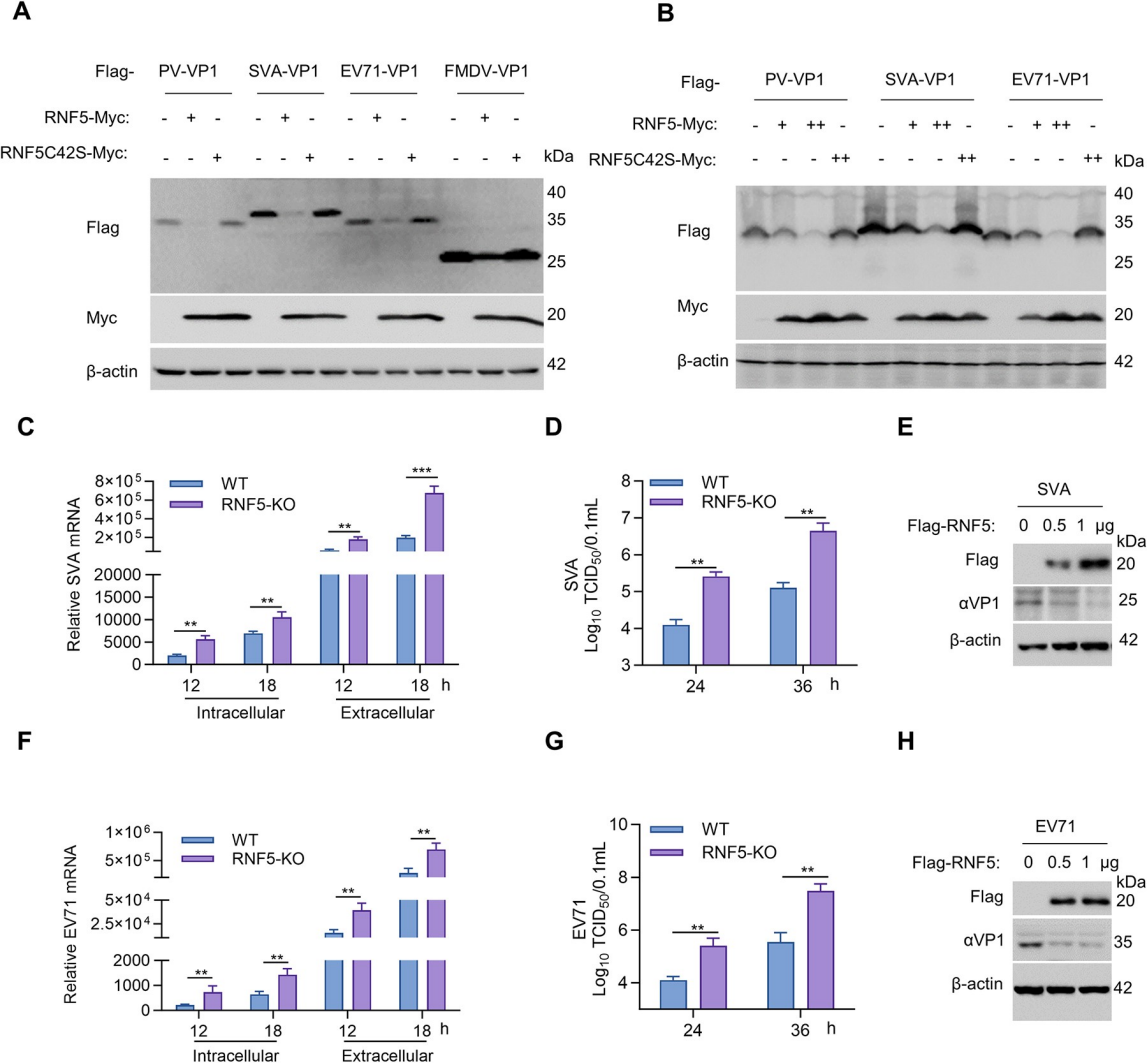
RNF5 inhibits several picornavirus replication by degrading VP1. (A) VP1 proteins of PV, SVA, and EV71 were degraded by RNF5. HEK293T cells were transfected with VP1 (1 μg) plasmids of PV, SVA, or EV71, along with Myc-RNF5 (1 μg) or Myc-RNF5C42S (1 μg) plasmids. The expression of Myc-RNF5 or Myc-RNF5C42S and Flag-tagged VP1 proteins was detected by immunoblot. (B) RNF5 induces the reduction of several picornavirus VP1 in a dose-dependent manner. HEK293T cells were transfected with VP1 (1 μg) plasmids of PV, SVA and EV71, along with increasing quantities of Myc-RNF5 (0, 1, 2 μg) or Myc-RNF5C42S (2 μg) plasmids. The expression of Flag-VP1 and Myc-RNF5 or Myc-RNF5C42S was detected by immunoblot. (C) Effects of RNF5 knockout on SVA replication. RNF5 knockout 293T cells were infected with SVA (MOI = 1.0) for the indicated times. The cell supernatant and precipitation were collected at 12 or 18 hpi, respectively. Intracellular and extracellular virus mRNA was determined by RT-PCR. (D) RNF5 knockout 293T cells were infected with SVA. At 24 or 36 hpi, the cell mixture, including supernatant and precipitation, was three freeze-thaw cycles, and then the viral titers were determined by TCID_50_ assay. (E) RNF5 inhibits the replication of endogenous SVA VP1 in a dose-dependent manner. IBRS-2 cells were transfected with RNF5 plasmids (0, 0.5, or 1 μg) for 24 h, and then infected with SVA for 12 h. The protein expression of SVA VP1 is detected by immunoblot. (F) Effects of RNF5 knockout on EV71 replication. RNF5 knockout 293T cells were infected with EV71 (MOI = 1.0) for the indicated times. The cell supernatant and precipitation were collected at 12 or 18 hpi, respectively. Intracellular and extracellular virus mRNA was determined by RT-PCR. (G) RNF5 knockout 293T cells were infected with EV71. At 24 or 36 hpi, the cell mixture, including supernatant and precipitation, was three freeze-thaw cycles, and then the viral titers were determined by TCID_50_ assay. (H) RNF5 inhibits the replication of endogenous EV71 VP1 in a dose-dependent manner. 293T cells were transfected with RNF5 plasmids (0, 0.5, or 1 μg) for 24 h, and then infected with EV71 for 12 h. The protein expression of EV71 VP1 is detected by immunoblot. Graphs show mean ± SD (n = 2 technical replicates, n = 3 biological replicates in C-G) from one representative experiment. Data were analyzed by two-way ANOVA, followed by sidak’s multiple comparisons test (C, D, F and G). **P<0.01; ***P<0.001.

FMDV is a positive-sense, single-stranded RNA virus, classified within the picornaviridae family. We have added FMDV to the list of viruses for which RNF5 acts as a host limiting factor, and RNF5 may also be involved with certain other picornaviruses. To investigate the antiviral effect of RNF5 on picornaviridae family, eukaryotic expression plasmids containing the VP1 gene from Poliovirus (PV), Senecavirus A (SVA), or Enterovirus 71 (EV71) were co-transfected into HEK293T cells along with RNF5 or RNF5C42S plasmids, respectively. Immunoblot analysis revealed that overexpression of RNF5 led to the degradation of VP1 in PV, SVA, and EV71, whereas RNF5C42S did not exhibit this effect (Fig 8A). Subsequent dose-dependent experiments confirmed these findings, showing that RNF5 degraded VP1 proteins in a dose-dependent manner (Fig 8B). The IFA results showed that RNF5 inhibits SVA-GFP replication in a dose-dependent manner (S5A Fig). Furthermore, the absence of RNF5 significantly enhanced viral mRNA levels at 12 and 18 hours post-infection (hpi), regardless of whether the viral RNA was located intracellularly or extracellularly (Fig 8C). Consistently, the viral titers in RNF5-KO cells exhibited an approximate 1.33 log_10_ TCID_50_/0.1 mL increase (equivalent to around a 21.3-fold rise) at 24 hpi, and a 1.36 log_10_ TCID_50_/0.1 mL elevation (corresponding to approximately a 22.9-fold increase) at 36 hpi (Fig 8D). Meanwhile, RNF5 also exhibited a dose-dependent inhibitory effect on endogenous SVA VP1 protein (Fig 8E).

To investigate the impact of RNF5 on EV71, RNF5-KO cells or WT cell were infected with EV71, and the intracellular and extracellular virus production was determined by RT-PCR. Whether it is intracellular or extracellular viral RNA, RNF5 deficiency also significantly promotes viral mRNA levels at 12 and 18 hpi (Fig 8F). The viral titer in RNF5-KO cells showed a ~1.4 log_10_ TCID_50_/0.1 mL (corresponding to ~25.1-fold) and ~1.44 log_10_ TCID_50_/0.1 mL (corresponding to ~27.5-fold) increase at 24 and 36 hpi, respectively (Fig 8G). Meanwhile, RNF5 also exhibited a dose-dependent inhibitory effect on endogenous EV71 VP1 (Fig 8H). Altogether, these findings suggest that RNF5 has a spectral antiviral effect on several picornaviruses by targeting VP1 for degradation through its E3 ubiquitin ligase activity.

## Discussion

Viruses are known to exploit the ubiquitin-proteasome system for their replication [30,31], but recent research indicates that certain components of this system, particularly E3 ligases, can act as intrinsic antiviral factors [32]. For example, TRIM22 has demonstrated broad-spectrum antiviral activity against various viruses such as encephalomyocarditis virus (EMCV), influenza A virus (IAV), hepatitis C virus (HCV), hepatitis B virus (HBV), and human immunodeficiency virus (HIV) [33–37]. Similarly, RNF114 has been identified as an antiviral protein against classical swine fever virus (CSFV) by targeting the NS4B protein for degradation [16]. Other examples include RNF125, which suppresses HIV replication [38], TRIM25, which inhibits infectious bursal disease virus (IBDV) proliferation [39], TRIM56, which shows antiviral properties against Bovine Viral Diarrhea Virus (BVDV) [40], and TRIM32, which restricts Influenza A virus by ubiquitinating the PB1 polymerase [41]. Additionally, TRIM52 has been found to impede Japanese Encephalitis Virus (JEV) replication by targeting the viral NS2A protein [42]. These antiviral TRIM proteins, which belong to the RING-type E3 ligase protein family, likely represent only a fraction of potential unexplored antiviral effectors within this group. Here, we discovered that RNF5 exhibits antiviral effects against FMDV replication. RNF5, an E3 ubiquitin ligase involved in regulating protein stability and clearance in various cellular processes [43,44], has been shown to negatively regulate virus-induced type I interferons by targeting MITA and VISA for ubiquitination and degradation, thereby inhibiting the cellular antiviral response [45,46]. Here, RNF5 has been found to directly interact with the VP1 protein, leading to its degradation and exerting direct antiviral effects.

The study revealed that RNF5 facilitates the degradation of VP1 via the ubiquitin proteasome pathway. Ubiquitination is a well-known process that regulates protein levels by targeting substrates for degradation through the ubiquitin-protease system (UPS) [47]. This posttranslational modification mechanism involves enzymes such as E1, E2, and E3, which attach ubiquitin molecules to target proteins, thereby controlling various cellular processes [48]. E3 ubiquitin ligases, which confer specificity to the ubiquitination process, are numerous compared to E1 and E2 enzymes [49]. The fate of ubiquitinated proteins is determined by the type of ubiquitin chains formed on the modified residue [50,51]. While K48 and K63 linkages are associated with proteasomal degradation and DNA repair, respectively, the functions of non-canonical polyUb chains (linked via K6, K11, K27, K29, and K33) are less understood and are currently under investigation. For instance, K6-linked chains are involved in DNA repair processes [52], while K11-polyUb may play roles in cell cycle regulation and ERAD [53]. K33-polyubiquitination influences T-cell receptor function and actin stabilization, among other functions [54,55]. K29-polyUbs are associated with growth pathways and mRNA stability regulation [56,57]. K27-linked chains are linked to mitochondrial damage and innate immunity regulation [58]. In the study, RNF5 was found to promote K48-linked polyubiquitination of VP1, leading to its degradation by the proteasome. The FMDV leader protein (L^pro^), while functioning as a deubiquitinase, is classified as a non-structural protein that is involved in the initial phases of the viral lifecycle. During the later stages of viral particle assembly, only the structural proteins VP1-VP4 come together to form a protomer, which then assembles into a pentamer with five protomers, and ultimately, 12 pentamers create a symmetrical icosahedral structure. Inside the capsid, there is a single-stranded positive-sense RNA. Consequently, the L^pro^ produced during the early phase of viral infection does not get incorporated into the virus particles. Thus, despite the L protein’s role as a deubiquitinase, it may not influence the ubiquitination and degradation of VP1 by RNF5 [59].

The E3 ubiquitin ligase enzyme alters the lysine residue of the target protein via ubiquitination. For example, the lysine residue at position 150 of the STING protein is a primary site for the attachment of a K63-linked ubiquitin chain mediated by TRIM56 [60]. Another instance is the lysine residue at position 104 of the nonstructural protein 3 (NS3) of Dengue Virus (DENV), which serves as a site for ubiquitination by TRIM69 [61]. Following viral infection, TRIM31 interacts with MAVS and facilitates K63-linked polyubiquitination at lysine residues 10, 311, and 461 of MAVS [62]. In this context, FMDV VP1 protein contains nine lysine residues, with lysine 200 being identified as a site for ubiquitination by RNF5. The inhibitory effect of RNF5 on the replication of the rO-WT virus was significant, while no impact was observed on the rO-VP1K200R virus, underscoring the importance of lysine 200 in VP1 degradation by RNF5. Through reverse genetics technology, it was demonstrated that the rO-VP1K200R mutant virus led to reduced survival rate in suckling mice. However, we do not present evidence indicating whether the VP1K200R mutation will affect the mortality of cloven-hoofed animals.

Furthermore, RNF5 not only demonstrates a notable antiviral effect at the cellular level but also confers protection to mice against FMDV, indicating its potential utility in molecular drug for disease resistance. The new small molecule, inh-2, which inhibits RNF5, led to a notable rescue of F508del-CFTR in both immortalized and primary bronchial epithelial cells from CF patients who are homozygous for the F508del mutation. In contrast, Analog-1, identified as a RNF5 activator due to its biological effects, negated the impact of inh-2 in the same cell models [63]. Notably, the RNF5 activator Analog-1 was found to effectively suppress FMDV replication in infection models utilizing cultured cells and mice, positioning it as a promising agent against FMDV-induced infections.

The function of RNF5 in various viruses is becoming clearer. Recent research has shown that RNF5 is involved in controlling the replication of SARS-CoV-2 and Kaposi sarcoma-associated herpesvirus (KSHV) [64]. In our research, we discovered that RNF5 inhibits several other picornaviruses, including PV, SVA, and EV71.

**Fig 9:**
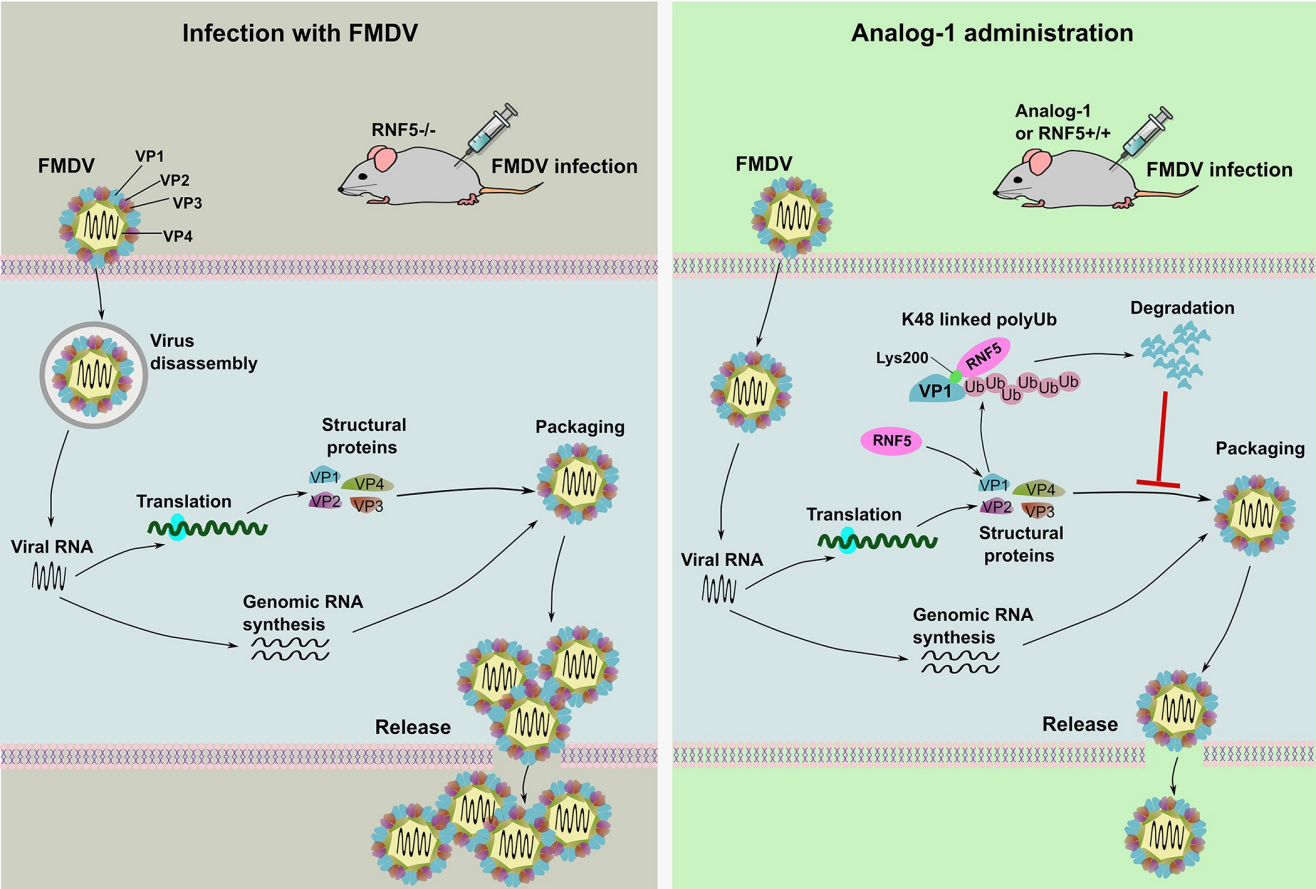
A model for RNF5’s anti-FMDV action. This study identifies the E3 ubiquitin-ligase RNF5 as a host factor that interacts with the FMDV VP1 protein to inhibit viral replication. Mutagenesis identified VP1 K200 as the site of ubiquitination by RNF5. A recombinant virus harboring the VP1 K200R mutation replicated to higher titers and exhibited increased pathogenicity in suckling mice. Mice lacking RNF5 exhibit greater histophathological damage to tissues, viral titers and higher mortality following FMDV infection. Treatment of cells with Analog-1 an agonist of RNF5 reduces FMDV replication in cells and i.p. administration to mice protects from FMDV disease. Our results identify RNF5-mediated VP1 protein degradation as a potential therapeutic strategy to treat infections caused by FMDV and related viruses.

In summary, this research findings demonstrate a significant inhibitory effect of RNF5 on FMDV replication. The presence of Cysteine at position 42 within RNF5, which confers E3 ubiquitin ligase activity, is identified as crucial for the antiviral function of RNF5. It was observed that RNF5 primarily influences virion formation during the FMDV lifecycle. Specifically, the interaction between RNF5 and VP1 leads to the K48-linked polyubiquitination of VP1 at Lys200. Experimental manipulation through reverse genetics revealed that mutations at Lys200 disrupted the ubiquitination and subsequent degradation of VP1, resulting in enhanced FMDV replication both *in vitro* and *in vivo* (Fig 5). Notably, RNF5 demonstrates a pronounced antiviral effect in both *in vitro* and *in vivo* settings (Figs 2 and 6), with the RNF5 activator Analog-1 proving to be effective in inhibiting FMDV replication in infection models utilizing cultured cells and mice, thereby positioning it as a potential therapeutic agent against FMDV infections (Fig 7). Furthermore, RNF5 was found to degrade several picornavirus VP1 proteins through its E3 ligase activity (Fig 8). These findings establish RNF5 as a novel broad-spectrum anti-picornavirus host factor that acts through K48-linked ubiquitination and degradation of VP1, offering potential utility in the prognosis of FMDV infections (Fig 9).

## Materials and methods

### Ethics statements

All animals were managed in adherence to established animal welfare protocols in accordance with the Animal Ethics Procedures and Guidelines of the People’s Republic of China. All experiments involving live FMDV were performed in the National Foot-and-Mouth Disease Reference Laboratory (ABSL-3), Lanzhou Veterinary Research Institute, Chinese Academy of Agricultural Sciences. The laboratory was accredited by China National Accreditation Service for Conformity Assessment and approved by the Ministry of Agriculture and Rural Affairs of the People’s Republic of China. This study followed the guidelines approved by the Gansu Animal Experiments Inspectorate and the Gansu Ethical Review Committee (Licence no. SYXK (GAN) 2010–003). Approval for all mouse studies was obtained from the Animal Ethics Committee of Lanzhou Veterinary Research Institute, Chinese Academy of Agricultural Sciences, under the approval number LVRIAEC-2024-053.

### Cells and viruses

Porcine kidney cell lines (PK-15, IBRS-2) and baby hamster kidney-21 (BHK-21) cells were cultured in minimum essential medium (MEM, Gibco, USA), while human embryonic kidney 293T (HEK293T) cells were cultured in Dulbecco modified Eagle medium (DMEM, Gibco, USA). The RNF5-KO-293T cell line was generously provided by Q. Zhu from Lanzhou Veterinary Research Institute, Lanzhou, China. The culture media for all cell lines were supplemented with 10% fetal bovine serum (FBS), 1% streptomycin (0.2 mg/mL), and penicillin (200 U/mL). The cells were maintained at 37°C with 5% CO_2_. The viral challenge was conducted using FMDV type O/BY/CHA/2010 and O/HN/CHA/2012 strain.

### Reagents and antibodies

The MG132 was purchased from Merck & Co (Germany). The NH_4_Cl and benzyloxy carbony (Cbz)-Val-Ala-Asp (OMe)-fluoromethylketone (Z-VAD-FMK) were purchased from Sigma-Aldrich (USA). The Analog-1 (STT-00001164) was purchased from Shanghai Yubo Biotechnology Co.,LTD. Antibodies against HA (Biolegend, Cat #901513), Flag (Sigma-Aldrich, Cat #F1804), Myc (Sigma-Aldrich, Cat #M5546), β-actin (Sigma-Aldrich, Cat #A5441), RNF5 (Abbkine, Cat #ABP60221), EV71 VP1 antibody (Bioss, Cat #bs-2297R), Mouse anti-goat IgG/Alexa Fluor 594 (Invitrogen, Cat #A11005), Rabbit anti-goat IgG/Alexa Fluor 488 (Invitrogen, Cat #A11008), Rabbit anti-goat IgG/Alexa Fluor 594 (Invitrogen, Cat #A11037), Mouse anti-goat IgG/Alexa Fluor 488 (Invitrogen, Cat #A28175), Ubiquitin antibody (Cell signaling Technology (CST), Cat #3933), K48-linkage specific polyubiquitin antibody (CST, Cat #4289) and K63-linkage specific polyubiquitin (D7A11) antibody (CST, Cat #5621) were purchased from the indicated manufacturers. Guinea pig anti-FMDV positive serum and mouse anti-VP1/VP2/VP3 antibody were obtained from the Lanzhou Veterinary Research Institute (LVRI). The SVA VP1 antibody was generously provided by Shichong Han from Henan Agricultural University, Zhengzhou, China.

### Plasmids

The VP1, RNF5, RNF5 (1–90), RNF5 (90–180), and RNF81 genes were cloned into the pcDNA3.1/myc-His A vector (Invitrogen, USA) to obtain HA/Flag-VP1, Myc/Flag-RNF5, Myc-RNF5-N, Myc-RNF5-C, and Flag-RNF81, respectively. The HA-tagged mutant VP1 proteins K41R, K46R, K95R, K109R, K133R, K167R, K179R, K200R, or K208R (lysine residue was replaced with arginine) and Myc-RNF5C42S constructs were generated by site-directed mutagenesis PCR. The pCMV-HA-Ub wild-type (WT) and mutants (K6, K11, K27, K29, K33, K48, K63, K48R, K63R) were obtained from BioZY Co., LTD. The PCR primer pairs were shown in S1 Table. All constructed plasmids were analyzed and verified by DNA sequencing.

### Coimmunoprecipitation assay (Co-IP) and Immunoblot analysis

HEK293T cells were cultured in 10-cm^2^ dishes, and the monolayer cells were co-transfected with various specified plasmids. The transfected cells were then cultured and lysed in 1 mL of lysis buffer composed of 20 mM Tris pH7.5, 150 mM NaCl, 1% Triton X-100, 1 mM EDTA, 10 mg/mL Aprotinin, 10 mg/mL Leupeptin, and 1 mM PMSF. Subsequently, each sample underwent incubation with 0.5 mg of an appropriate antibody and 40 μL of protein G-Sepharose in 20% ethanol (GE Healthcare) for a duration of 12 hours. The sepharose beads were subjected to three washes with 1 mL of lysis buffer containing 500 mM NaCl. The resulting precipitates were then subjected to analysis through an immunoblot assay. In the immunoblot procedure, the target proteins were separated via SDS-PAGE and transferred onto an Immobilon-P membrane (Millipore, USA). Following this, the membrane was blocked and exposed to suitable primary and secondary antibodies. The visualization of the antibody-antigen complexes was achieved using enhanced chemiluminescence detection reagents (Thermo, USA).

### Immunofluorescence microscopy

PK-15 or BHK-21 cells were cultured on Nunc glass-bottom dishes for a duration of 24 hours and subsequently exposed to FMDV at an MOI of 0.5 for 8 or 12 hours at 37°C. Following this, the cells were fixed using 4% paraformaldehyde for 30 minutes and permeabilized with 0.1% Triton X-100 for 15 minutes. The cells were then subjected to incubation in 5% BSA at 4°C for 4 h, followed by treatment with the appropriate primary antibody and either Alexa Fluor 488 or 594-conjugated secondary antibody. Subsequently, the cellular images were captured utilizing a laser-scanning confocal microscope (LSCM, Leica SP8, Solms, Germany).

### CRISPR/Cas9 knockout

The gRNA design was guided by recommendations provided on the Zhang laboratory website (http://crispr.mit.edu/). To create the gRNA expression plasmid, complementary oligonucleotides encoding gRNA-252 (5’-CCGAAGGGCCAAACCGCGAG-3’) and gRNA-304 (5’-TATATGTCTGGAGACTGCTC-3’) were annealed and inserted into BbsI (NEB) restriction sites within the PX459 vector (Addgene#62988). Subsequently, PX459-gRNA plasmids were transfected into PK-15 or IBRS-2 cells using Polyplus jetPRIME transfection (jetPRIME114-15). Following transfection, cells were treated with puromycin (3 μg/mL) for 3 days. The genomic region encompassing the gRNA target site was amplified via PCR utilizing specific primers (forward: 5’-attagaaaaccctgaggcctgccc-3’; reverse: 5’-taaggtggctcgggtctcatctgc-3’), and the resulting PCR products were purified and sequenced. The efficacy of knockout was validated through immunoblot analysis, with wild-type (WT) PK-15 or IBRS-2 cells serving as controls.

### Adsorption and internalization assay

In the FMDV adsorption experiment, RNF5-KO or wild-type (WT) cells were exposed to FMDV at an MOI of 10 and incubated at 4°C for 1 hour. After the adsorption process, any unattached viruses were eliminated by rinsing with ice-cold PBS, and the quantity of cell-bound viral RNA was determined using RT-PCR. For the FMDV internalization study, cells were treated with FMDV at an MOI of 10 at 4°C for 1 hour. Subsequently, unattached viruses were washed away with ice-cold PBS, and the cells were then transferred to 37°C for 1 hour to facilitate viral internalization. Viruses that did not penetrate the cells were eliminated using PBS containing proteinase K, and the levels of internalized viral RNA was assessed through RT-PCR.

### TCID_50_ titration

BHK-21 cells were used to titrate the released infectious virus. The infected cells were harvested at the indicated time post infection, and the titers were determined in terms of 50% tissue infection dose (TCID_50_)/100 μL by using the Reed-Muench method [65].

### Reverse genetics

The strategy for construction of the plasmid used to produce the recombinant virus was as described previously by our laboratory [66]. At 48 h post-transfection, the cell supernatants were harvested by centrifugation at 6000×g for 10 min at 4°C and passaged in BHK-21 cells for 4 times. The recovered viruses were named as rO-VP1K200R mutant virus and rO-WT virus, which were stored in the National Foot-and-Mouth Disease Reference Laboratory (ABSL-3), Lanzhou Veterinary Research Institute, Chinese Academy of Agricultural Sciences following the standard protocols and biosafety regulations provided by the Institutional Biosafety Committee. Both of viruses were amplified by PCR using the primers used previously (66), and the PCR products were sequenced by the amplified primers.

### Plaque assay

BHK-21 cell lines were plated in six-well cell culture dishes a day prior to infection. FMDV was cultured in MEM with an inoculum volume of 200 μL per well. Following a one-hour adsorption period, the inoculum was aspirated, and the cells were covered with a mixture of 50% gum tragacanth and 50% 2×minimal essential medium supplemented with 2% fetal bovine serum. Subsequently, the plates were incubated for 48 hours, fixed using a solution of acetone and methanol in a 1:1 ratio, and stained with crystal violet (Amresco, USA).

### RNA extraction and RT-PCR

Total RNA was isolated utilizing TRIzol Reagent (Invitrogen, USA), followed by cDNA synthesis from the extracted RNA samples employing M-MLV reverse transcriptase (Promega, USA) and random hexamer primers (TaKaRa, Japan). The resulting cDNA served as the template for assessing the expression levels of FMDV RNA and host cellular mRNA. RT-PCR was conducted using the Mx3005P QPCR system (Agilent Technologies, USA) and SYBR Premix ExTaq reagents (TaKaRa, Japan) to quantify RNA levels, with the specific primers detailed in S2 Table. The glyceraldehyde-3-phosphate dehydrogenase (GAPDH) gene was utilized as an internal reference control. The relative mRNA expression was determined through the comparative cycle threshold (2^-ΔΔCT^) method.

### RNA interference (RNAi)

The small interfering RNA (siRNA) utilized in the RNA interference (RNAi) experiment was synthetically produced by GenePharma in Shanghai, China. The silencing of endogenous RNF5 was achieved through the transfection of RNF5 siRNA (5’-CCUUGUUGAUUUAAUUUAAUU-3’, 5’-GGCAUUCAGACCUAUAGUAAU-3’, or 5’-GCGCGACCUUCGAAUGUAAUA-3’) into PK-15 cells using Polyplus jetPRIME transfection reagent. The silencing of endogenous RNF81 was achieved through the transfection of RNF81 siRNA (5’- CGAGAAACUCAGAAAUAAATT-3’, 5’-UGGCCAACAUGGUGGAUAATT-3’, or 5’-CCGAAAACGAGGAGAGAUUTT-3’) into PK-15 cells. A non-targeting siRNA (NC siRNA: 5’-UUCUCCGAACGUGUCACGUTT-3’) served as a negative control in the study.

### Establishment of RNF5 knockout mice using the CRISPR/Cas9 system

RNF5^-/-^ and RNF5^+/+^ mice on the C57BL/6J background were generated using CRISPR/Cas9 technology. The strategy for construction of the targeting vector was illustrated in Fig 6A. Exon 2–6 and part of intron of RNF5 gene were targeted by two specific gRNAs, which led to the deletion of exon 2–6 (1861 bp) of RNF5 coding sequence. Genotyping was performed by PCR with the following primers: F1: 5’-TTATGAGGAGGAATGGGAAATGGG-3’, R1: 5’-GCAGAGCTGAATCAGTCA GAGGA -3’, and R2: 5’-CAACATCCTCCACACATTTGATCC-3’. Amplification of the WT allele produced a 539 bp fragment, and amplification of the disrupted allele produced a 462 bp fragment.

### Cell viability assay

The Cell Counting Kit-8 (CCK-8) from Yeasen was employed to evaluate cell viability in this study. Cells were plated in 96-well plates and incubated for 6–8 hours before being treated with 10 μL of CCK-8 solution as per the manufacturer’s guidelines. Cell viability was assessed by measuring the absorbance of the cells at OD_450_ nm after a 2-hour incubation period.

### Mice infection

In each group, age- and sex-matched 3-day-old suckling mice were subcutaneously inoculated in the neck with 0.2 mL of PBS, rO-WT, or rO-VP1K200R (20LD_50_) in PBS. The survival rates of all groups (n = 6 per groups) were monitored for a period of 7 days. Three mice per group were euthanized on day 3 post-infection to check for lesions in the lungs.

In the infection experiment involving RNF5^-/-^ mice, age- and sex-matched 3-day-old RNF5^-/-^ and RNF5^+/+^ suckling mice were challenged with a subcutaneous injection of 20LD_50_ of FMDV in 0.2 mL of PBS. The survival rates of all groups (n = 6 per groups) were observed for 8 days. Five mice per group were euthanized on day 3 post-infection to check for virus replication in the muscle, heart, liver, spleen, lung, kidney, and duodenum. Three mice per group were euthanized on day 3 post-infection to check for lesions in the heart, liver, spleen, lung, kidney, and duodenum.

In the Analog-1 assay, mice were divided into four groups, two groups received 10 mg/kg Analog-1 treatment seven times every two days via intramuscular injection, followed by infection with FMDV/O/HN/CHA/2012 at a dosage of 30LD_50_ through intraperitoneal challenge. Another two negative control groups were not treated with Analog-1 or infected with FMDV. The survival rates of all groups (n = 7 per groups) were observed for 8 days. Three mice per group were euthanized on day 3 post-infection to check for lesions in the lungs.

### Histological assessment

Following euthanasia of the mice, their tissues were gathered and promptly preserved in 10% neutral-buffered formalin. The preserved tissues were then embedded in paraffin, sectioned, and subjected to staining with hematoxylin and eosin (H&E) for subsequent histopathological examinations, as conducted by Wuhan Servicebio Technology Co., Ltd.

### Gel filtration analysis

Wild-type and RNF5-KO cells were infected FMDV for 12 h. Subsequently, cells were collected and lysed in lysis buffer (20 mM Tris-HCl, pH 7.4, 150 mM NaCl, 1 mM EDTA, 1% NP-40, supplemented with protease and phosphatase inhibitors). Cell lysates were centrifuged at 12000 g, 4°C for 30 min. Subsequently, cell supernatants were filtered (0.22 μm filter; Millipore) and subjected to gel filtration chromatography using Superdex 200 Increase 10/300 GL column and separation buffer (50 mM Tris, pH 7.4, 150 mM NaCl). Fraction collector collected 1 mL per fraction for 12 fractions after balancing with separation buffer of 1/5 column volume. A gel filtration standard (151–1901; Bio-Rad) was also run to calibrate the fractions.

### Statistical analysis

All data were presented as means±SD and analyzed using GraphPad Prism software (version 10.1.0). Individual statistical tests are specified within the figure legends. For data with two groups, unpaired students’t tests were used under the assumption of normality. Data with more than two groups were analyzed by analysis of variance (ANOVA) under assumption of normality. In general, at least three independent biological replicates (n) were carried out for each experiment. Data were reproduced in independent experiments as indicated in the legends. Significant differences are denoted in the figures as follows: *P<0.05; **P<0.01; ***P<0.001; ****P<0.0001; n.s., indicating no statistical significance.

## Supporting information

S1 Fig RNF5 inhibits FMDV replication. (A) Cell viability assays of transient transfection of Flag-RNF5 or Flag-RNF81. The Cell Counting Kit-8 (CCK-8) from Yeasen was employed to evaluate cell viability in this study. (B and C) Evaluation of the efficiency of NC or RNF5 siRNA in silencing RNF5 expression. PK-15 cells seeded into 6-well plates were transfected with 150 nM NC or RNF5 siRNA (si-1, si-2, si-3) for 36 h. The knockdown efficiency was then determined by RT-PCR (B) and immunoblot analysis (C). (D) Evaluation of the efficiency of NC or RNF81 siRNA in silencing RNF81 expression. PK-15 cells seeded into 6-well plates were transfected with 150 nM NC or RNF81 siRNA (si-1, si-2, si-3) for 36 h. The knockdown efficiency was then determined by RT-PCR. (E) Schematic chromatogram representation of gRNA targeting at the pRNF5 genomic region. PAM sequences are underlined and highlighted in green. sgRNA targeting sites are underlined and highlighted in red. (F) The alignment of the RNF5 genomic nucleotide sequence of the published RNF5 reference sequence and the RNF5-WT, PK-RNF5-KO-1, and PK-RNF5-KO-2 sequences using LaserGene software. The red box indicates the regions that were mutated. (G) Confirmation of the genome editing by Sanger sequencing the PCR amplicon from the RNF5 genome of the PK-RNF5-KO cell lines. (H) The alignment of the RNF5 genomic nucleotide sequence of the published RNF5 reference sequence and the RNF5-WT, IBRS-RNF5-KO-1, and IBRS-RNF5-KO-2 sequences using LaserGene software. The red box indicates the regions that were mutated. (I) Confirmation of the genome editing by Sanger sequencing the PCR amplicon from the RNF5 genome of the IBRS-RNF5-KO cell lines. (J and K) Cell viability of PK-RNF5-KO or IBRS-RNF5-KO cell lines stably knockout for pRNF5. The Cell Counting Kit-8 (CCK-8) from Yeasen was employed to evaluate cell viability. (L) Immunofluorescence analysis of FMDV at 0.5 MOI for 8 h in PK-RNF5-WT and PK-RNF5-KO-1 cells. The viral proteins were detected using guinea pig anti-FMDV serum (Green). Graphs show mean ± SD (n = 3 technical replicates in A and D, n = 2 technical replicates in B, J and K, n = 3 biological replicates) from one representative experiment. Data were analyzed by two-way ANOVA (A) or one-way ANOVA (B, D, J and K), followed by Dunnett’s multiple comparisons test. *P<0.05; **P<0.01; ****P<0.0001; n.s., indicating no statistical significance.
(TIF)

S2 Fig RNF5 degrades FMDV VP1 protein and affects virion assembly. (A) Relative fold-change in the abundance of VP1 protein in Fig 3A data was determined by densitometric analysis using ImageJ Launcher analysis. (B and C) RNF5 induces the reduction of VP1 in a dose-dependent manner. HEK293T cells were transfected with Flag-VP1 plasmid and increased quantities of Myc-RNF5 plasmids. The expression of Flag-VP1 and Myc-RNF5 was detected by immunoblot. Relative fold-change in the abundance of VP1 protein was determined by densitometric analysis using ImageJ Launcher analysis. (D) The overexpression of RNF5 did not lead to a significant reduction in VP1 mRNA levels. HEK293T cells were transfected with VP1 plasmid and increased quantities of Myc-RNF5 plasmids. The expression of VP1 mRNA was detected by RT-PCR. (E) Knockout of RNF5 did not affect FMDV attachment. The WT or RNF5-KO cells were infected with FMDV at an MOI of 10 and cultured at 4°C for 1 h. After adsorption, the unbound viruses were extensively washed away with ice-cold PBS. The cell-bound FMDV virions were quantified by RT-PCR. (F) Knockout of RNF5 did not affect FMDV internalization. The WT or RNF5-KO cells were infected with FMDV at an MOI of 10 and incubated at 4°C for 1 h. The unbound FMDV virions were washed away with ice-cold PBS, and the cells were switched to 37°C for 1 h. After washes, the internalized FMDV virions were quantified by RT-PCR. (G) Schematic illustration of bicistronic FMDV IRES construct. (H) Endogenous RNF5 does not affect FMDV IRES-driven translation. PK-15 WT or RNF5-KO cells were transfected with the bicistronic construct FMDV-IRES or vector plasmids. At 36 h posttransfection, the Rluc and Fluc activities were determined. (I and J) The effect of RNF5 on vRNA synthesis. PK-15 WT or RNF5-KO cells were infected with 1 MOI FMDV at the indicated time. Positive (+vRNA) (I) or negative (-vRNA) (J) viral RNA was quantified by RT-PCR. Graphs show mean ± SD (n = 2 technical replicates, n = 3 biological replicates) from one representative experiment. Data were analyzed by one-way ANOVA with Dunnett’s multiple comparisons test (A, C, D, E and F), or two-way ANOVA with sidak’s multiple comparisons test (H, I and J). **P<0.01; ***P<0.001; n.s., indicating no statistical significance.
(TIF)

S3 Fig RNF5 mediated the ubiquitination of VP1. (A and B) The effect of endogenous RNF5 on VP2, and VP3 ubiquitination in FMDV-infected cells. RNF5-KO cells or WT cells were transfected with HA-Ub plasmid with or without FMDV (MOI = 5) for 8 h in the presence of MG132 (20 μM), and were subjected to IP with anti-VP2 or anti-VP3 antibody. Membranes blotted with antibodies against HA, VP2, VP3 and RNF5. (C) Schematic representation of a panel of Ub mutants. (D) The effect of endogenous RNF5 on VP1 ubiquitination in FMDV-infected cells. RNF5-KO cells or WT cells were transfected with Ub plasmid with or without FMDV (MOI = 5) for 8 h in the presence of MG132 (20 μM), and were subjected to IP with anti-VP1 antibody. Membranes blotted with antibodies against HA, VP1, and RNF5. (E) Relative fold-change in the abundance of VP1 protein in Fig 5B data was determined by densitometric analysis using ImageJ Launcher analysis. Graphs show mean ± SD (n = 2 technical replicates). Data were analyzed by two-way ANOVA with sidak’s multiple comparisons test (E). **P<0.01; n.s., indicating no statistical significance.
(TIF)

S4 Fig RNF5 pharmacological activator Analog-1 alleviates the pathogenic effects of FMDV in a mouse model. (A-C) PK-15 cells were exposed to varying concentrations of Analog-1 (0, 25, 50, and 100 μM) and subsequently infected with rO-VP1K200R at 0.1 MOI. Following a 24-hour post-infection period, the levels of viral RNA, viral protein, and viral titers were assessed through RT-PCR (A), immunoblot (B), and TCID_50_ (C), respectively. Graphs show mean ± SD (n = 2 technical replicates, n = 3 biological replicates) from one representative experiment. Data were analyzed by one-way ANOVA with Dunnett’s multiple comparisons test (A and C).
(TIF)

S5 Fig RNF5 inhibits several picornavirus replication by degrading VP1. (A) RNF5 inhibits SVA replication in a dose-dependent manner. IBRS-2 cells were seeded into 6-well plates and transfected with RNF5 plasmids of different concentrations. After transfection for 24 hours, SVA-GFP was infected, and fluorescence was observed and photographed after 12 hours of infection.
(TIF)

S1 File Biosafety and biosecurity measures. (DOCX)

S1 Table The PCR primer pairs used in this study. (DOCX)

S2 Table Primers used for mRNA quantification. (DOCX)

S1 Data Data that underlies this paper. Excel spreadsheet containing, in separate sheets, the underlying numerical data for Figs 2A, 2C–2D, 2F, 2H, 2J, 2L, 2N, 3D–3E, 3J, 5H, 6D–6E, 7A–7B, 7D, 8C–8D, 8F–8G, S1A, S1B, S1D, S2A, S2C–S2F, S2H–S2J, S3E, S4A and S4C.
(XLSX)
