# Supplementary figures and images for: RING finger protein 5 is a key anti-FMDV host factor through inhibition of virion assembly
Source: PLoS Pathog. 2025 Jan 17;21(1):e1012848. doi: 10.1371/journal.ppat.1012848 (PMC11741381; doi:10.1371/journal.ppat.1012848)

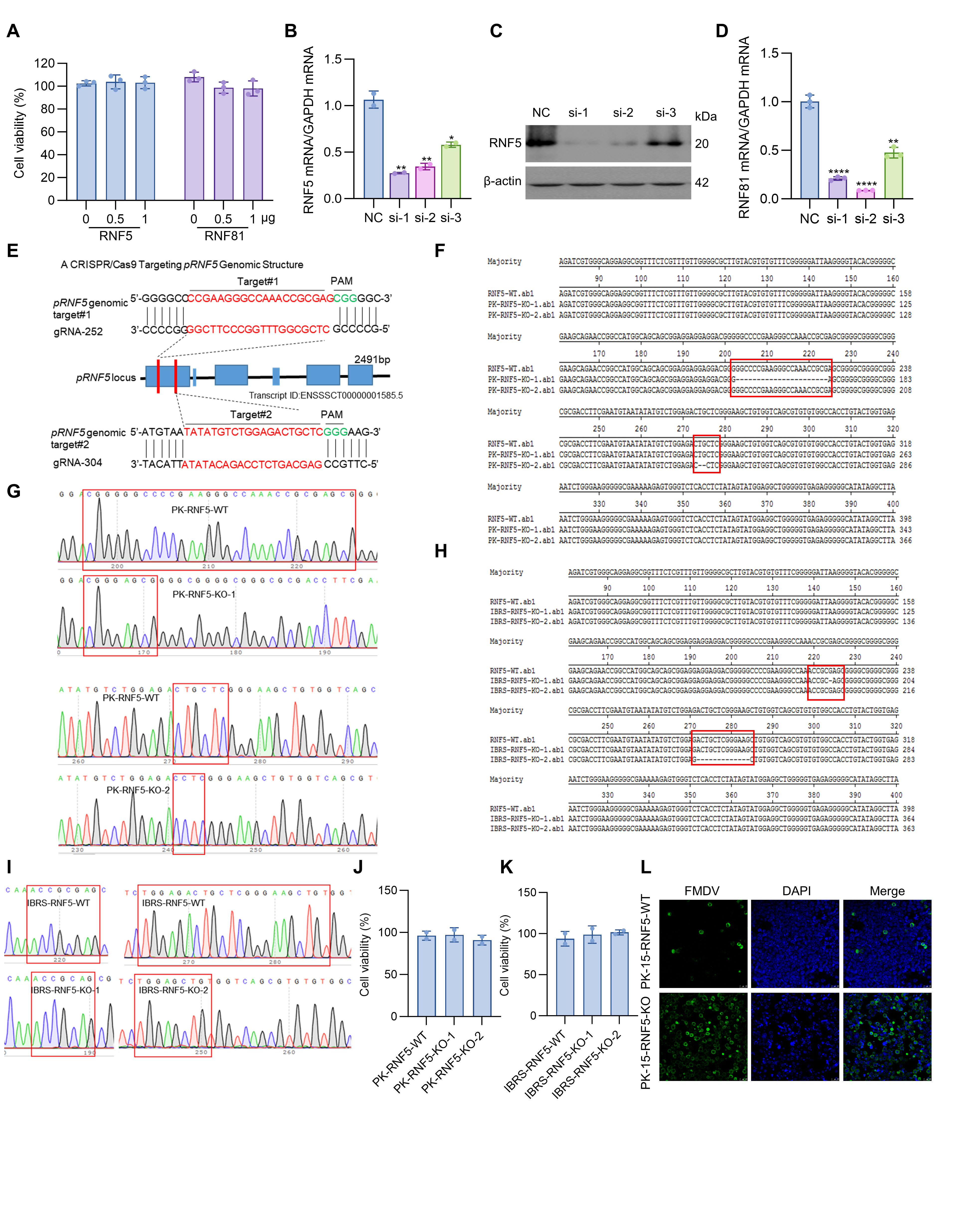

Supplement: S1 Fig — (A) Cell viability assays of transient transfection of Flag-RNF5 or Flag-RNF81. The Cell Counting Kit-8 (CCK-8) from Yeasen was employed to evaluate cell viability in this study. (B and C) Evaluation of the efficiency of NC or RNF5 siRNA in silencing RNF5 expression. PK-15 cells seeded into 6-well plates were transfected with 150 nM NC or RNF5 siRNA (si-1, si-2, si-3) for 36 h. The knockdown efficiency was then determined by RT-PCR (B) and immunoblot analysis (C). (D) Evaluation of the efficiency of NC or RNF81 siRNA in silencing RNF81 expression. PK-15 cells seeded into 6-well plates were transfected with 150 nM NC or RNF81 siRNA (si-1, si-2, si-3) for 36 h. The knockdown efficiency was then determined by RT-PCR. (E) Schematic chromatogram representation of gRNA targeting at the pRNF5 genomic region. PAM sequences are underlined and highlighted in green. sgRNA targeting sites are underlined and highlighted in red. (F) The alignment of the RNF5 genomic nucleotide sequence of the published RNF5 reference sequence and the RNF5-WT, PK-RNF5-KO-1, and PK-RNF5-KO-2 sequences using LaserGene software. The red box indicates the regions that were mutated. (G) Confirmation of the genome editing by Sanger sequencing the PCR amplicon from the RNF5 genome of the PK-RNF5-KO cell lines. (H) The alignment of the RNF5 genomic nucleotide sequence of the published RNF5 reference sequence and the RNF5-WT, IBRS-RNF5-KO-1, and IBRS-RNF5-KO-2 sequences using LaserGene software. The red box indicates the regions that were mutated. (I) Confirmation of the genome editing by Sanger sequencing the PCR amplicon from the RNF5 genome of the IBRS-RNF5-KO cell lines. (J and K) Cell viability of PK-RNF5-KO or IBRS-RNF5-KO cell lines stably knockout for pRNF5. The Cell Counting Kit-8 (CCK-8) from Yeasen was employed to evaluate cell viability. (L) Immunofluorescence analysis of FMDV at 0.5 MOI for 8 h in PK-RNF5-WT and PK-RNF5-KO-1 cells. The viral proteins were detected using guinea pig anti-FMDV se [file ppat.1012848.s001.tif]

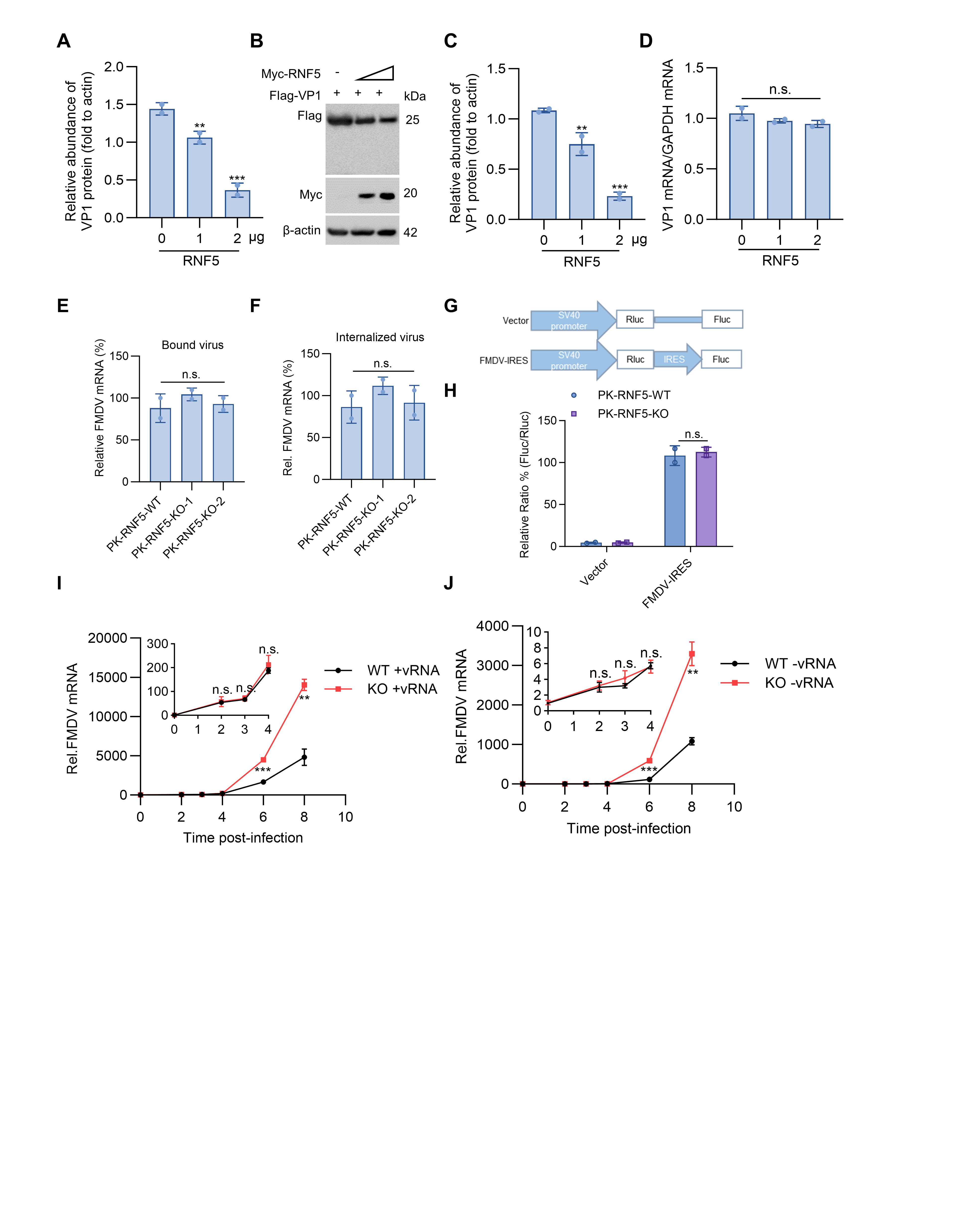

Supplement: S2 Fig — (A) Relative fold-change in the abundance of VP1 protein in Fig 3A data was determined by densitometric analysis using ImageJ Launcher analysis. (B and C) RNF5 induces the reduction of VP1 in a dose-dependent manner. HEK293T cells were transfected with Flag-VP1 plasmid and increased quantities of Myc-RNF5 plasmids. The expression of Flag-VP1 and Myc-RNF5 was detected by immunoblot. Relative fold-change in the abundance of VP1 protein was determined by densitometric analysis using ImageJ Launcher analysis. (D) The overexpression of RNF5 did not lead to a significant reduction in VP1 mRNA levels. HEK293T cells were transfected with VP1 plasmid and increased quantities of Myc-RNF5 plasmids. The expression of VP1 mRNA was detected by RT-PCR. (E) Knockout of RNF5 did not affect FMDV attachment. The WT or RNF5-KO cells were infected with FMDV at an MOI of 10 and cultured at 4°C for 1 h. After adsorption, the unbound viruses were extensively washed away with ice-cold PBS. The cell-bound FMDV virions were quantified by RT-PCR. (F) Knockout of RNF5 did not affect FMDV internalization. The WT or RNF5-KO cells were infected with FMDV at an MOI of 10 and incubated at 4°C for 1 h. The unbound FMDV virions were washed away with ice-cold PBS, and the cells were switched to 37°C for 1 h. After washes, the internalized FMDV virions were quantified by RT-PCR. (G) Schematic illustration of bicistronic FMDV IRES construct. (H) Endogenous RNF5 does not affect FMDV IRES-driven translation. PK-15 WT or RNF5-KO cells were transfected with the bicistronic construct FMDV-IRES or vector plasmids. At 36 h posttransfection, the Rluc and Fluc activities were determined. (I and J) The effect of RNF5 on vRNA synthesis. PK-15 WT or RNF5-KO cells were infected with 1 MOI FMDV at the indicated time. Positive (+vRNA) (I) or negative (-vRNA) (J) viral RNA was quantified by RT-PCR. Graphs show mean ± SD (n = 2 technical replicates, n = 3 biological replicates) from one representative experiment. Data we [file ppat.1012848.s002.tif]

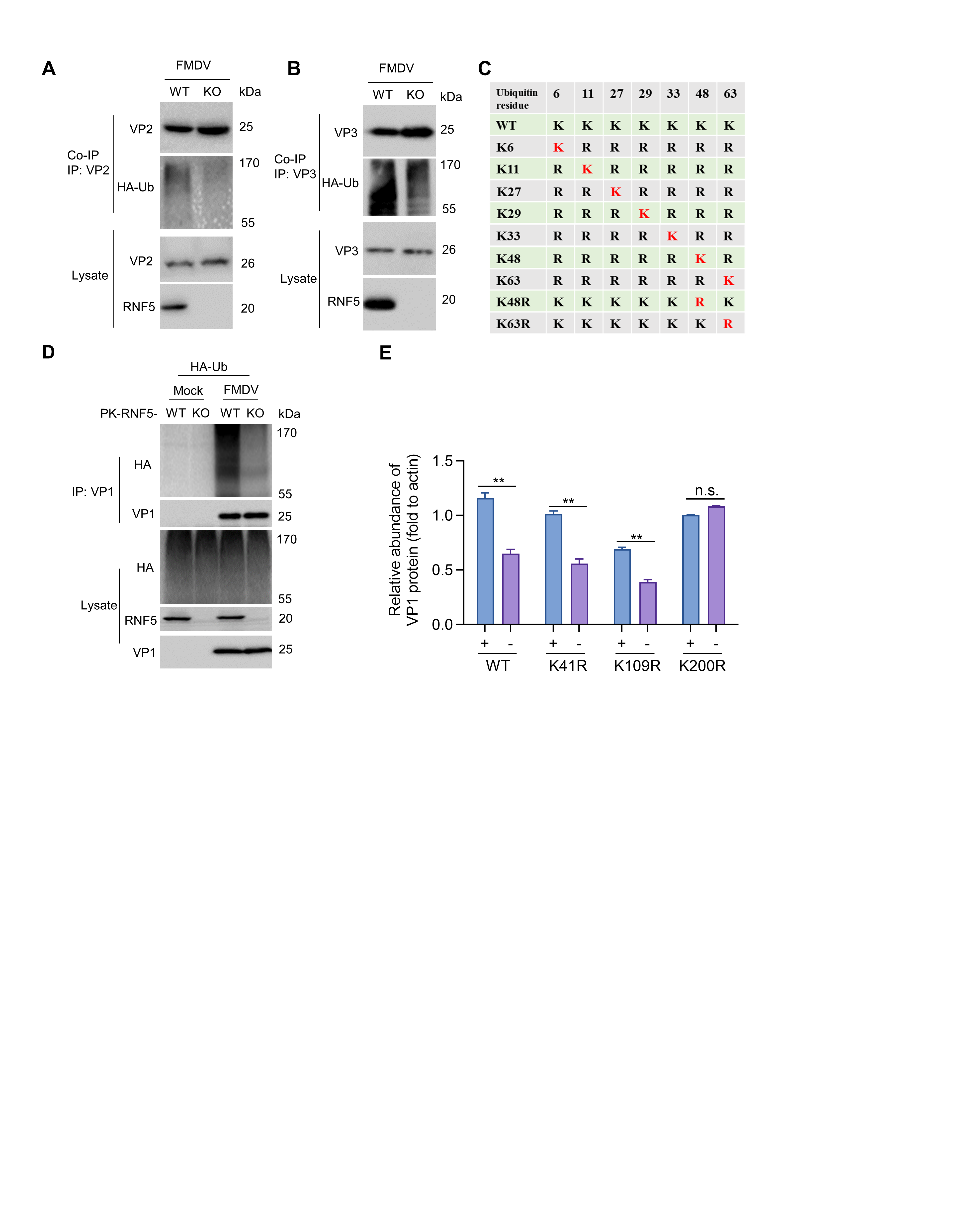

Supplement: S3 Fig — (A and B) The effect of endogenous RNF5 on VP2, and VP3 ubiquitination in FMDV-infected cells. RNF5-KO cells or WT cells were transfected with HA-Ub plasmid with or without FMDV (MOI = 5) for 8 h in the presence of MG132 (20 μM), and were subjected to IP with anti-VP2 or anti-VP3 antibody. Membranes blotted with antibodies against HA, VP2, VP3 and RNF5. (C) Schematic representation of a panel of Ub mutants. (D) The effect of endogenous RNF5 on VP1 ubiquitination in FMDV-infected cells. RNF5-KO cells or WT cells were transfected with Ub plasmid with or without FMDV (MOI = 5) for 8 h in the presence of MG132 (20 μM), and were subjected to IP with anti-VP1 antibody. Membranes blotted with antibodies against HA, VP1, and RNF5. (E) Relative fold-change in the abundance of VP1 protein in Fig 5B data was determined by densitometric analysis using ImageJ Launcher analysis. Graphs show mean ± SD (n = 2 technical replicates). Data were analyzed by two-way ANOVA with sidak’s multiple comparisons test (E). **P<0.01; n.s., indicating no statistical significance. (TIF) [file ppat.1012848.s003.tif]

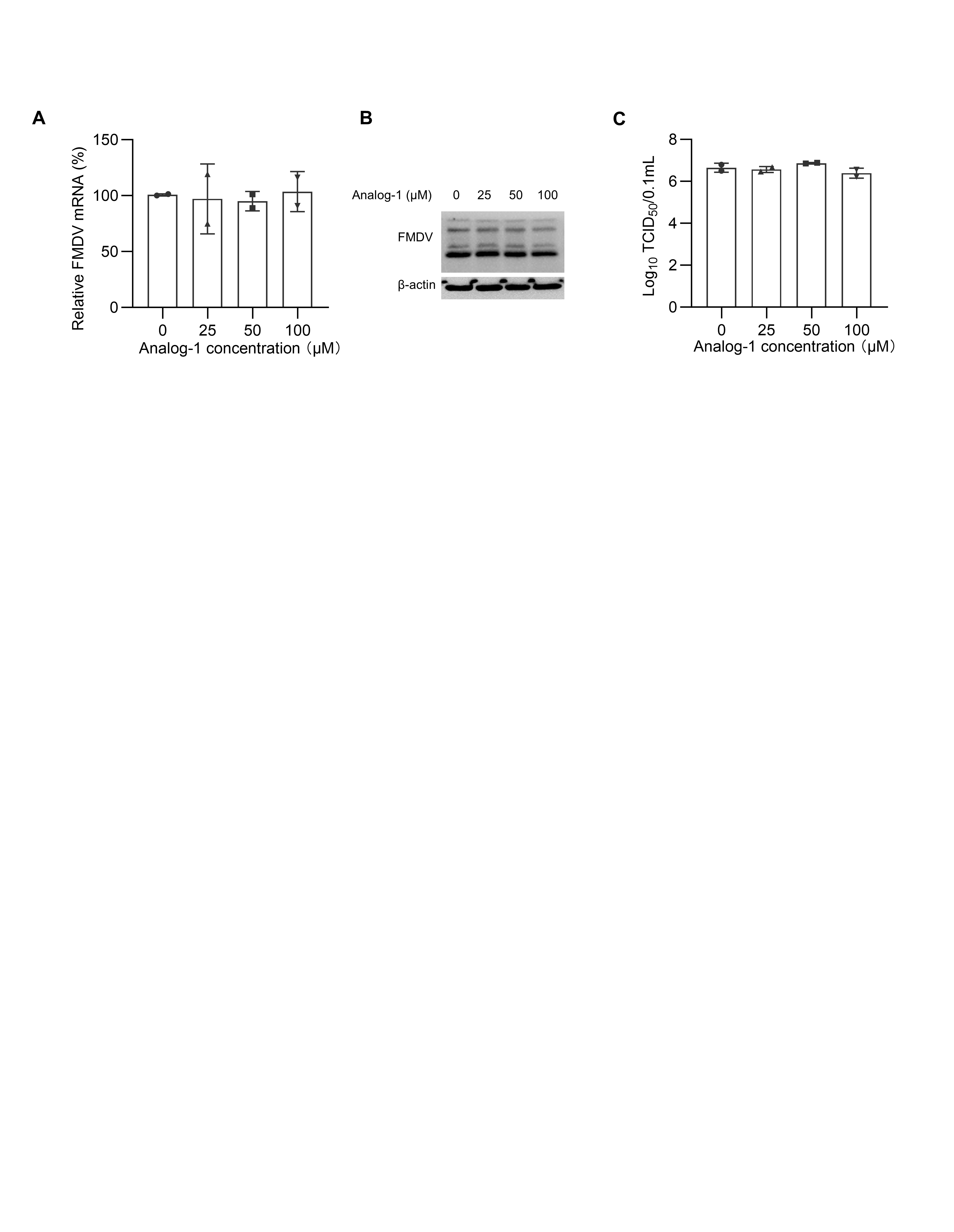

Supplement: S4 Fig — (A-C) PK-15 cells were exposed to varying concentrations of Analog-1 (0, 25, 50, and 100 μM) and subsequently infected with rO-VP1K200R at 0.1 MOI. Following a 24-hour post-infection period, the levels of viral RNA, viral protein, and viral titers were assessed through RT-PCR (A), immunoblot (B), and TCID50 (C), respectively. Graphs show mean ± SD (n = 2 technical replicates, n = 3 biological replicates) from one representative experiment. Data were analyzed by one-way ANOVA with Dunnett’s multiple comparisons test (A and C). (TIF) [file ppat.1012848.s004.tif]

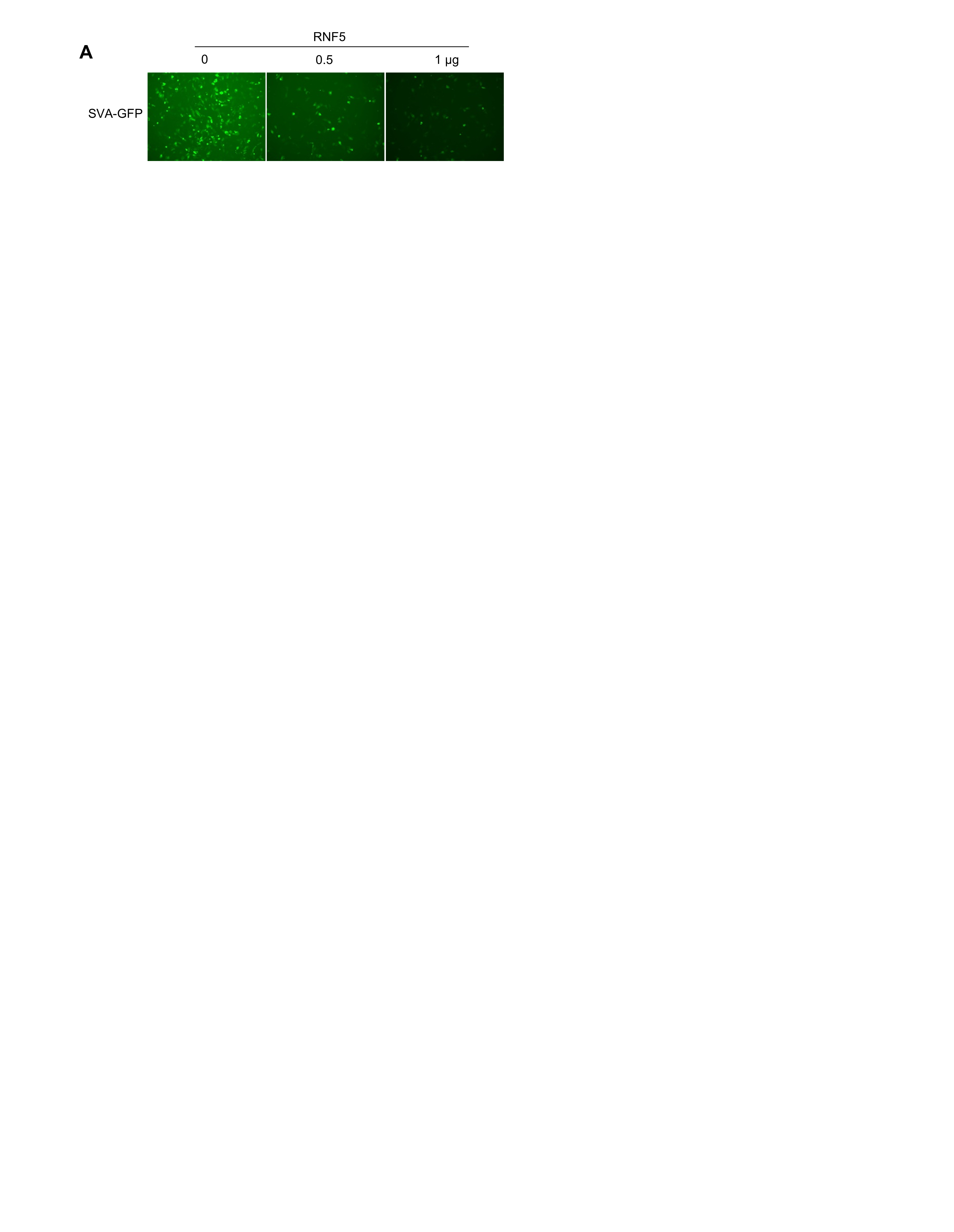

Supplement: S5 Fig — (A) RNF5 inhibits SVA replication in a dose-dependent manner. IBRS-2 cells were seeded into 6-well plates and transfected with RNF5 plasmids of different concentrations. After transfection for 24 hours, SVA-GFP was infected, and fluorescence was observed and photographed after 12 hours of infection. (TIF) [file ppat.1012848.s005.tif]
