# Supplementary material for: RING finger protein 5 is a key anti-FMDV host factor through inhibition of virion assembly
Source: PLoS Pathog. 2025 Jan 17;21(1):e1012848. doi: 10.1371/journal.ppat.1012848 (PMC11741381; doi:10.1371/journal.ppat.1012848)
Supplement: S1 File — (DOCX) [file ppat.1012848.s006.docx]

**Supplementary Materials**

**Biosafety and biosecurity measures**

According to biosafety laws and regulations ([1](#_ENREF_1)), all experiments involving live FMDV were conducted within the animal biosafety level 3 (ABSL-3) facility in the Lanzhou Veterinary Research Institute (LVRI), Chinese Academy of Agricultural Sciences (CAAS), which was accredited by China National Accreditation Service for Conformity Assessment and approved by the Ministry of Agriculture and Rural Affairs of the People’s Republic of China. The ABSL-3 facility consists of negatively pressurized rooms in which all in vivo and in vitro experimental work is conducted in class 3 isolators or class 3 biosafety cabinets, which operate under negative pressure.

All activities inside the ABSL-3 labs are monitored by a trained guard via video camera. Only authorized personnel that have received appropriate training can access the ABSL-3 facility. For animal handling in the facilities, experienced personnel work in pairs. Our staff wear N95 respirators, and disposable coveralls; they shower on exiting the facility. The facility is secured by appropriate procedures approved by the LVRI institutional biosafety officers. All facilities, procedures, training records, safety drills, and inventory records are subject to periodic inspections and ongoing oversight by the institutional biosafety officers who consult frequently with the facility managers. The research program, procedures, occupational health plan, security and facilities are reviewed annually by a Ministry of Agriculture official.

According to the regulations set forth by the Ministry of Agriculture and Rural Affairs regarding the preservation and management of animal pathogenic microorganisms (viruses)([2](#_ENREF_2)), the National Foot-and-Mouth Disease Reference Laboratory (ABSL-3) of Lanzhou Veterinary Research Institute, Chinese Academy of Agricultural Sciences, serves as the preservation unit for FMDV. This laboratory has established a comprehensive technical data archive that details the names, numbers, quantities, sources, categories of pathogenic microorganisms, main characteristics, preservation methods, and other relevant information about the preserved bacteria (viruses) and samples.

Additionally, the laboratory has developed emergency plans for handling laboratory safety incidents. In the event of theft, robbery, loss, leakage, or infection of stored bacterial (toxic) species or samples by laboratory personnel, timely reporting and activation of contingency plans must be executed in accordance with the "Regulations on Biological Safety Management of Pathogenic Microbial Laboratories, and appropriate measures shall be implemented.

In any of the following circumstances, the preservation institution shall convene experts to discuss and propose recommendations for the destruction of bacterial (toxic) strains or samples: (1) those that should be destroyed according to national regulations; (2) evidence indicates that it has either lost its biological activity or become contaminated, rendering it unsuitable for continued use; (3) without any additional preservation value.

To restrict access to these strains, preservation institutions shall provide bacterial toxin strains or samples in accordance with the following regulations: (1) verify the approval documents required for conducting experiments involving these microorganisms; (2) verify the approval number document for animal drug production; (3) verify the certificate issued by the unit where the laboratory is located.

**Reference:**

1. Notice of the Ministry of Agriculture on Further Standardizing the Approval of Experimental Activities on Pathogenic Microorganisms in Highly Pathogenic Animals 2008. Available from: <http://www.moa.gov.cn/gk/tzgg_1/tz/200812/t20081225_1196297.htm>.

2. Preservation and management of animal pathogenic microorganisms (viruses) 2022;https://[www.gov.cn/zhengce/2022-01/07/content_5721410.htm](http://www.gov.cn/zhengce/2022-01/07/content_5721410.htm).
