# Supplementary material for: RING finger protein 5 is a key anti-FMDV host factor through inhibition of virion assembly
Source: PLoS Pathog. 2025 Jan 17;21(1):e1012848. doi: 10.1371/journal.ppat.1012848 (PMC11741381; doi:10.1371/journal.ppat.1012848)
Supplement: S1 Table — (DOCX) [file ppat.1012848.s007.docx]

**Table S1：The PCR primer pairs used in this study.**

| Primers | Sequences (5' to 3') | Target gene |
| --- | --- | --- |
| VP1-NheI-F | CGTCTA*GCTAGC*ATGACTACCACCACTGG | FMDV VP1 gene |
| VP1-BamHI-R | CGC*GGATCC*CAAAGTCTGTTTCTCAGGTG |  |
| RNF5-NheI-F | CGTCTA*GCTAGC*ATGGCAGCAGCGGAGGAGG | RNF5 gene |
| RNF5-BamHI-R | CGC*GGATCC*AATACTGAGCAGCCAGAAAA |  |
| RNF5-N-NheI-F | CGTCTA*GCTAGC*ATGGCAGCAGCGGAGGAGGAGG | RNF5-N gene |
| RNF5-N-BamHI-R | CGC*GGATCC*GGGATCCTGGGGCTTCTGGCTCC |  |
| RNF5-C-NheI-F | CGTCTA*GCTAGC*AGATTGAAAACTCCACCCCGCCC | RNF5-C gene |
| RNF5-C-BamHI-R | CGC*GGATCC*AATACTGAGCAGCCAGAAAAA |  |

The italics represent enzymes.
