# Supplementary material for: RING finger protein 5 is a key anti-FMDV host factor through inhibition of virion assembly
Source: PLoS Pathog. 2025 Jan 17;21(1):e1012848. doi: 10.1371/journal.ppat.1012848 (PMC11741381; doi:10.1371/journal.ppat.1012848)
Supplement: S2 Table — (DOCX) [file ppat.1012848.s008.docx]

**S2 Table：Primers used for mRNA quantification.**

| Primers | Sequences (5' to 3') |
| --- | --- |
| GAPDH Forward | ACATGGCCTCCAAGGAGTAAGA |
| GAPDH Reverse | GATCGAGTTGGGGCTGTGACT |
| RNF5 Forward | GCTTTCACTTCTCGTTTGG |
| RNF5 Reverse | AAATGGCTCGTGGGTATT |
| RNF81 Forward | CTTCTACAACATCAGTGA |
| RNF81 Reverse | ATTGAAACCAGGATTGAA |
| FMDV Forward | CACTGGTGACAGGCTAAGG |
| FMDV Reverse | CCCTTCTCAGATTCCGAGT |
| VP1 Forward | TTGAGAACTACGGAGGAG |
| VP1 Reverse | TACGAACCTGTCGAGAAC |
| SVA Forward | AGAATTTGGAAGCCATGCTCT |
| SVA Reverse | GAGCCAACATAGARACAGATTGC |
| EV71 Forward | GCTCTATAGGAGATAGTGTGAGTAGGG |
| EV71 Reverse | ATGACTGCTCACCTGCGTGTT |
